# Adverse events of trimetroprim-sulphonamide treatment of cats and dogs: a systematic review

**DOI:** 10.1007/s11259-026-11143-1

**Published:** 2026-03-25

**Authors:** Carl Ekstrand, Mia Hedlund, Lena Pelander, Karolina Scahill

**Affiliations:** 1 Department of Animal Biosciences, Swedish University of Agricultural Sciences, Uppsala, Sweden; 2 Department of Clinical Sciences, Swedish University of Agricultural Sciences, Uppsala, Sweden; 3 International Veterinary Evidence-based Guidelines Centre, Department of Veterinary Clinical Sciences, University of Copenhagen, Frederiksberg C, Denmark; 4 Evidensia Södra Djursjukhuset Kungens Kurva, Stockholm, Sweden

**Keywords:** Potentiated sulphonamides, Pharmacovigilance, Companion animals, Breed predisposition Sulfonamide toxicity

## Abstract

Potentiated sulphonamides (TMS) are recommended first-line antimicrobials in veterinary medicine, yet their use may be limited by concerns about adverse events (AEs). This systematic review aimed to evaluate the frequency, nature, and potential risk factors for TMS-associated AEs in dogs and cats. Following Preferred Reporting Items for Systematic reviews and Meta-Analyses (PRISMA) reporting standards, 110 studies published between 1943 and 2024 were included, covering > 4200 treated animals. A pairwise meta-analysis of eight comparative studies did not suggest a clinically relevant increased risk of AEs with TMS compared to other antimicrobials in either cats or dogs. A proportional meta-analysis of 31 studies in dogs showed an estimated prevalence of mild (2.3%) and severe (0.2%) AE. AE were described in more detail in 80 studies. The most commonly described severe AE were keratoconjunctivitis sicca, immune-mediated polyarthritis, hepatic necrosis, and haematological disorders. Severe AEs were reported in 64 studies and 247 animals, while mild AEs were reported in 23 studies. The most frequently implicated combinations were sulfadiazine- or sulfamethoxazole-trimethoprim. Time to onset ranged from hours to weeks and appeared longer for severe reactions. AEs were more frequently reported after treatment durations exceeding seven days. In breed-specific analyses, Doberman Pinschers were overrepresented among cases with polyarthritis. These findings suggest that while TMS can cause serious AEs, the incidence is low, and judicious use may reduce reliance on critically important antibiotics.

**Supplementary Information:**

The online version contains supplementary material available at 10.1007/s11259-026-11143-1.

## Introduction

Potentiated sulfonamides (TMS) are combinations of a diaminopyrimidine (e.g. ormetoprim or trimethoprim) and a sulfonamide (e.g. sulfadiazine, sulfadimethoxine or sulfamethoxazole). The first sulfonamide has been dated to 1932, and in 1935 the first experimental and clinical effects were reported (Domagk 1957). Trimethoprim, which also acts as a sulfonamide potentiator, was discovered several decades later, with its first clinical use appear to have been reported in 1962 (Noall et al. 1962; Bushby and Hitchings 1968).

After oral administration, TMS are well absorbed and widely distributed to the tissues, including those protected by physiological barriers such as the cerebrospinal fluid and nervous tissues (Dowling 2025). However, there are pharmacokinetic differences between TMP and sulphonamides because TMP distributes to the tissue in larger extent (Frimodt-Möller et al., 1979, Sigel et al., 1981; Pohlenz-Zertuche et al., 1992). Elimination involves both hepatic metabolism and renal excretion in unchanged form, with TMP in general having a shorter plasma half-life than sulphonamides.

TMS act on a broad spectrum of bacteria, including both Gram-negative and Gram-positive organisms, by inhibiting sequential enzymatic steps in the incorporation of para-aminobenzoic acid into folic acid, thereby blocking folic acid biosynthesis (Dowling 2025). This broad antibacterial activity, together with the extensive tissue distribution of TMS, suggests they could be a feasible alternative for treating soft tissue infections caused by Gram-negative bacteria, such as pyelonephritis, prostatitis, and sepsis in dogs and cats.

The World Health Organization (WHO) has advocated that antibiotics considered critically important to human health—such as monobactams, lipopeptides, and carbapenems—should not be used in animals (Anonymous 2024a). Consistent with WHO recommendations, the European Medicines Agency has published guidelines for the use of antibiotics in veterinary medicine, also including substances that should be avoided, e.g. fluoroquinolones, and the designation of recommended first-line agents (Anonymous 2020). One example of first line antibiotics is TMS. However, adverse events (AE), such as immune-mediated disease, have been described in association with TMS treatment in cats and dogs (Noli et al. 1995; Trepanier et al. 2003). A perceived high risk of severe AE may contribute to the underutilization of TMS by veterinary practitioners, who instead may favour fluoroquinolones for the treatment of the aforementioned conditions. By increasing use of TMS in veterinary medicine, the use of critically important antimicrobials for humans, such as fluoroquinolones, could be minimized and help preserve the efficacy of those antibiotics. To address this issue, systematic collection and analysis of clinical data regarding adverse events associated with TMS is warranted. Accurate characterization of their frequency, severity, and clinical context is essential to enable robust risk assessments. Such evidence is crucial for informed clinical decision-making and may support more prudent antimicrobial use.

The primary aim of this systematic review is to assess the risk of AE associated with TMS treatment in dogs and cats. Secondary aims include characterizing the clinical presentation and severity of reported AE, as well as identifying potential predisposing factors such as breed, dose, treatment duration, alone or in comparison to other antibiotics.

## Materials and methods

### Protocol and registration

The study protocol was registered with Synthesizing Research for Animals & Food (SYREAF) (syreaf.org) in May 2025: https://syreaf.org/wp-content/uploads/2025/05/carl-ekstrand-publikation-tms-protocol.pdf. The protocol prospectively predefined the review questions, eligibility criteria, outcomes, and analytic approach. To facilitate readability in the present manuscript, key methodological elements are summarized below, while full details are available in the registered protocol, which is included as Supplementory file 1.There were no changes to the protocol. This systematic review adhered to the Preferred Reporting Items for Systematic reviews and Meta-Analyses (PRISMA) reporting guidelines. The PRISMA checklist is reported in Supplementary file 2.

### Objectives/research questions

Four research questions that were formulated in the Population, Intervention, Comparator and Outcome (PICO) framework (Table 1) were included in this systematic review. Two questions did not include a comparator but a context and aimed to characterize the outcome and report proportional risk. The PICO framework guided all stages of study selection, data extraction, and analysis and was applied a priori in accordance with the registered protocol.

**Table 1:** shows the PICOs (population, intervention, comparator and outcome) included in this systematic review. TMS = potentiated sulfonamides (e.g., sulfadiazine–trimethoprim or sulfamethoxazole–trimethoprim)

#	Population	Intervention	Comparator/context	Outcome
1	Cats and dogs	TMS	Other antimicrobials	Adverse events
2	Cats and dogs	TMS	Proportion	Adverse events
3	Cats and dogs	TMS	Outcome characterization	Adverse events
4	Cats and dogs	TMS long duration (> 7 days)	TMS short duration (≤ 7 days)	Adverse events

### Eligibility criteria

All studies that reported AEs of TMS and/or trials using TMS were included. No date restrictions were applied. Study design eligibility varied between the different PICOs (Table 2). For PICO 2 cohorts of TMS-treated animals were included only if ≥ 10 animals were enrolled to limit prevalence estimates from very small denominators while retaining informative zero-event cohorts with adequate denominators. Studies reporting AEs were included regardless of size in PICO 1, 3 and 4.

**Table 2:** Shows eligibility criteria for each PICO. PICO 1required comparative designs (randomized controlled trials or controlled observational cohorts) comparing TMS with another antimicrobial. PICO 2 estimated prevalence; therefore, randomized and observational studies were included, and case series were eligible only when n≥10 to avoid unstable proportions from very small denominators. PICO 3 synthesized adverse event (AE) phenotypes and included any design that reported As. Abbreviations: TMS, potentiated sulfonamides; RCT, randomized controlled trial; AE, adverse event

PICO	Inclusion	Exclusion
1	Randomized controlled trials, controlled cohorts using TMS	Reviews, case reports, trials without controls, conference abstracts, editorial letters, other reports
2	Randomized controlled trials, observational studies, case series (≥10 animals) using TMS	Reviews, case reports, trials without controls, conference abstracts, editorial letters, other reports
3	Randomized controlled trials, all observational studies (controlled and uncontrolled), case reports, conference abstracts, editorial letters that report adverse events for TMS	Reviews (but references screened)
4	Randomized controlled trials, controlled cohorts using TMS and another antimicrobial	Reviews, case reports, conference abstracts, editorial letters, other reports
1,2,3,4	Experimental disease studies (disease conditions such as induced urinary tract infection).	Experimental studies and safety studies with exceptionally high doses that are not used in practice. All experimental studies on other species than cats and dogs
1,2,3,4	Swedish, English, German, Danish, Norwegian	Other languages

### Information sources and search strategy

Searches were conducted between 2024-10-24 and 2024-10-30 in the CABI databases: Cab abstracts, Medline, Web of Science Core Collection, PubMed and Scopus. Search strategies combined controlled vocabulary and free-text terms for cats and dogs with terms for trimethoprim–sulphonamide compounds and were adapted to each database. The full search strategies are provided in the registered protocol. Duplicate removal was performed in Endnote, according to the Karolinska Institutet Library’s method (https://kib.ki.se/node/1379. 387). Grey literature, i.e. non–peer-reviewed reports, guidelines, and policy documents, was not explored. The search strategy is reported in the protocol referenced above.

### Data management and selection process

Screening was done in the systematic review tool Covidence (www.covidence.org) in duplicate by two of 4 reviewers (CE, LP, MH, KS). Conflicts were resolved through discussion or by a third reviewer. Callibrations exercises were done both for screening and for data extraction where two reviewers independently screened an initial training set (approximately 10% of records), discussed disagreements, and refined inclusion/exclusion rules. A second small pilot confirmed agreement before proceeding.

#### Title and abstract screening

In the first level, the reviewers (CE, KS, LP) independently evaluated the protocols relevance by using the following screening question:

1. Does the abstract include TMS side effects or TMS treatment of cats and dogs?
  Yes= include, No= exclude, Uncertain: include for full text evaluation.

2. Is the abstract written in English, Swedish, Norwegian, Danish or German?
  Yes= include, No= exclude.

#### Full text screening

In the second level, the reviewers (CE, KS, LP, MH) independently evaluated the protocols relevance by using the following screening question:

1. Does the full-text report TMS-associated AE (any study design and sample size)? If AE are not reported, does it describe TMS treatment in at least 10 cats and dogs?
  Yes= include, No= exclude
2. Is the full text written in English, Swedish, Norwegian, Danish or German?
  Yes=include, No=exclude (if the abstract includes the relevant information in one of the included languages it can however be included)
3. Does the study include TMS dosages that are not used in practice (defined as over 15-30 mg/kg once or twice daily)?
  Yes=exclude, No=include

### Data collection process and items

Data was extracted into an Excel spreadsheet (https://office.microsoft.com/excel) independently by two of four reviewers (CE, KS, LP, MH). Conflicts were solved through discussion or by a third reviewer. The following data was extracted: 1) General information: first author, title, journal, year of publication, funding, country of study (the country of the corresponding author was chosen if the country is not disclosed, 2) Study design: RCT, observational, case report, retrospective/prospective, multi or single centre, experimental infection (yes/no), setting (shelter/owned animals/laboratory animals), 3) Treatment information: substance (intervention and comparator), total animals treated, total animals with AE, dose, duration of treatment, time to onset of AE (from start of treatment), 4) Adverse event information: hypersensitivity, KCS, non-hypersensitivity, mortality (due to AE), recovery, breed and sex of animals with hypersensitivity reaction and KCS.

#### Outcomes and prioritization

The primary outcome was AE. Adverse events were sub-categorized and defined as mild or severe (Table 3). Mortality due to AE was a separate outcome. All categorizations were applied as reported by the source studies to avoid *post hoc* reclassification and potential misclassification bias.

**Table 3:** Examples (non-exhaustive) of adverse events (AEs) used for severity categorization. Severe AEs were defined as life-threatening, requiring intensive treatment, associated with organ failure, or irreversible Mild AEs were defined as transient, self-limiting, or reversible without lasting sequelae (e.g., mild dermatologic reactions, transient swelling/urticaria, gastrointestinal signs, lethargy/mild stiffness, polyuria/polydipsia, reversible KCS). Examples reflect reported study terminology; events were classified by clinical presentation and reversibility, and we did not infer underlying mechanisms. Abbreviation: KCS, keratoconjunctivitis sicca

Severe	Mild
1. Immune-mediated disease (sometimes called hypersensitivity reactions) such as thrombocytopenia, pemphigus and polyarthritis.	1. Mild dermatological reactions
2. Anaphylactic chock	2. Transient swelling/urticaria
3. Hepatic necrosis, acute kidney injury	3. Gastrointestinal disease (diarrhoea, vomiting)
4. Irreversible KCS and other irreversible conditions	4. Lethargy/mild stiffness
	5. Polyuria/polydipsia
	6. Reversible KCS

### Data analysis and presentation of results

Data included in PICO 1 was synthesized descriptively and described in absolute numbers. A pairwise meta-analysis was be performed for PICO 1 by using the RevMan web tool (https://revman.cochrane.org) using the inverse variance method and a random effects analysis model and the restricted maximum-likelihood method for heterogeneity. The Wald-type method was used to calculate the 95% confidence intervals (CI). RevMan web’s signalling risk of bias questions based on the Cochranes’s risk of bias 2 tool (Sterne et al., 2019), were used to assess both randomized controlled trials and observational studies. The proportional meta-analysis was performed in the online tool PERSystMA app (https://persyst.group/persystma).

GRADEpro (https://www.gradepro.org/) was used to generate summary of findings tables and to calculate absolute effects for PICO 1. The Grading of Recommendations, Assessment, Development and Evaluation (GRADE) methodology was used to assess the certainty of evidence for PICO 1 (Guyatt et al. 2008). GRADE considers five domains: risk of bias (study limitations that could distort results), inconsistency (unexplained variability in results across studies), indirectness (differences between the available evidence and our PICO in population, intervention, comparator, or outcomes), imprecision (uncertainty around the effect estimate), and publication bias (selective publication or reporting). For imprecision, we judged whether 95% confidence intervals crossed prespecified absolute risk-difference thresholds for clinical importance (Table 4) and whether the accumulated sample size and number of events were adequate. Based on concerns in these domains, overall certainty was rated as high, moderate, low, or very low in accordance with GRADE guidance. Treatment duration was dichotomized a priori (< 7 days vs. ≥ 7 days) to enable structured comparison across heterogeneous study designs, as prespecified in the protocol. Clinical thresholds (Table 4) used to assess the imprecision domain was formulated by the present authors and based on their values and preferences (three veterinary clinicians (KS, LP, MH) and one veterinary pharmacologist (CE), three are also dog owners) without further stakeholder involvement (Schünemann et al. 2022). We refer to the recently upated GRADE book for further information about the GRADE methodology (Neumann et al., 2024).

**Table 4:** Clinical thresholds that were used to assess the imprecision domain. The numbers are number of adverse events per 1000 animals that were considered to be a small, moderate or large effect. The thresholds were chosen by the authors (three veterinary clinicians and one veterinary pharmacologist) and reflects their values and preferences

Adverse events	Small threshold (%)	Moderate threshold (%)	Large threshold (%)
Mild	100 (10)	200 (20)	500 (50)
Severe	50 (5)	75 (7.5)	100 (10)

## Results

**Fig. 1:**
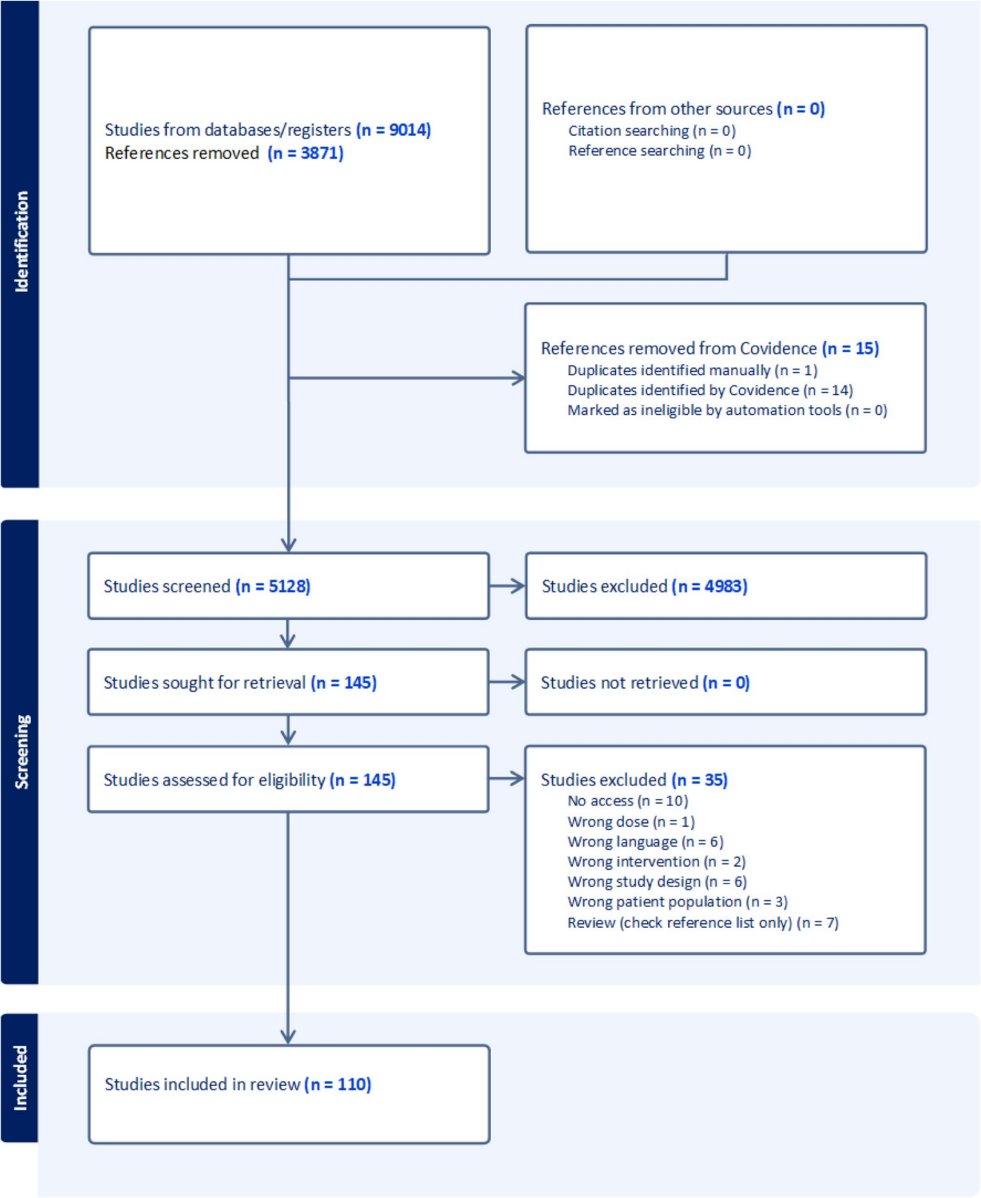
The PRISMA flowchart shows includes number of exclusions, inclusions and deduplications. *Karolinska Institute Library method is described and referenced in the main text. Created by the Covidence software (covidence.org)

**Fig. 2:**
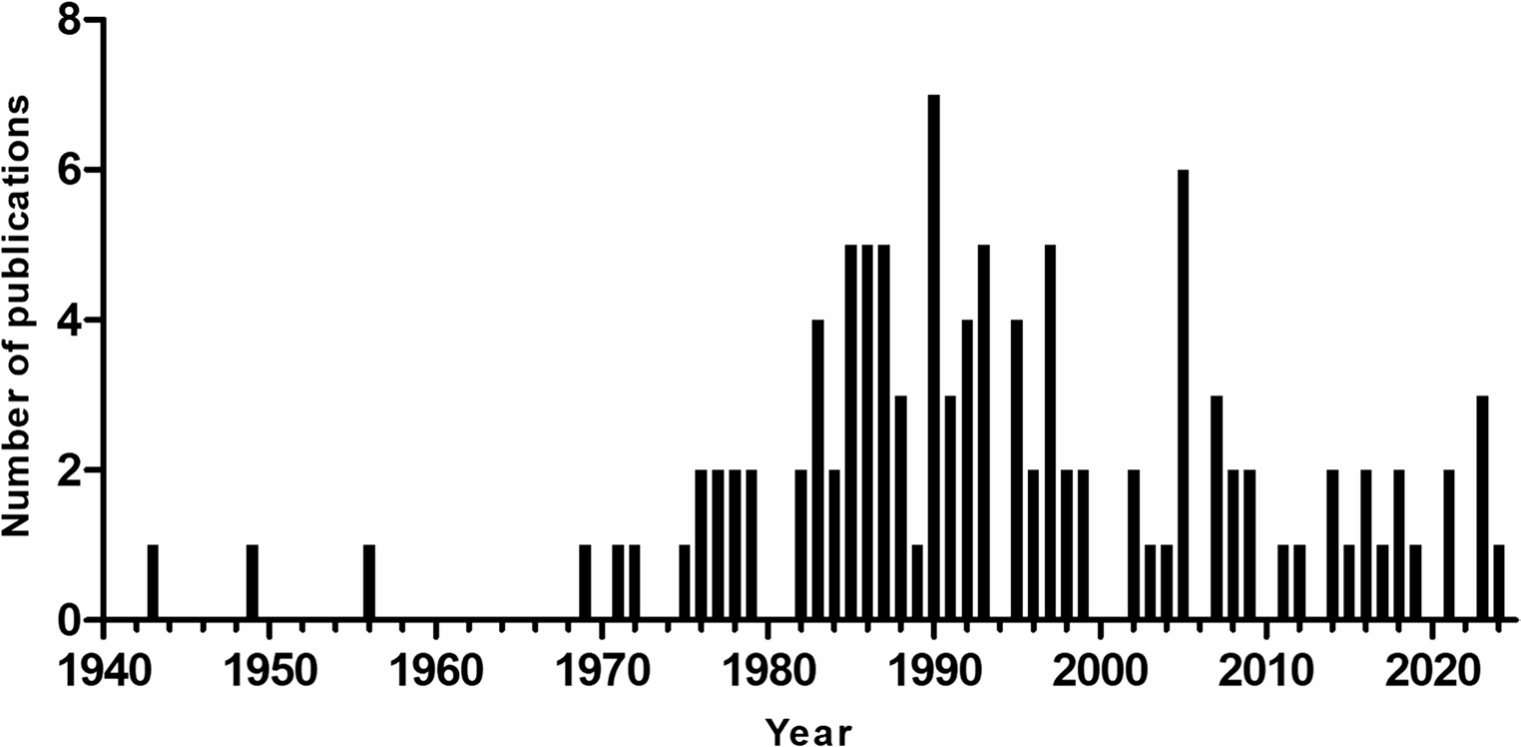
Year of publication for included papers

One hundred and ten studies met the inclusion criteria and were included in the present review. Eight publications were included in PICO 1 and 31 publications were included in PICO 2, 80 publications were included in PICO 3 and 58 publications were included in PICO 4 although no paper specifically investigated treatment duration. Some publications were included in multiple PICOs (Table 5). Number of search hits, duplicate removal and reasons for exclusion are reported in the PRISMA flowchart (Fig. 1). The 110 studies included in this review were published between 1943 and 2024 (Fig. 2). A majority (53/110, 48.2%) were from the US, followed by the UK and Germany (15/110, 13.6% and 7/110, 6.46%, respectively). Of the included studies, 36 (32.7%) were retrospective observational studies, 35 (31.8%) were case reports, 25 (22.7%) were prospective observational studies and 9 (8.2%) were randomized controlled trials. Five studies did not fit in any of these categories. Thirty-five (31.8%) of the studies were conducted at single centers, while 14 (12.7%) were conducted at multiple centers. The remaining 61 studies had no information about number of centers. Study characteristics are reported in further detail in Table 5.

**Table 5:** Study-level characteristics of all included reports. Columns list: first author and year, country, study design, study population (species/setting), disease/indication, TMS regimen (dose and interval) and, where applicable, comparator regimen, followed by the number of adverse events (AEs) attributed to TMS classified as severe or mild within each study. A value of 0 indicates the study explicitly reported no events; NR indicates not reported; NNS indicates AEs were mentioned but counts were not specified. Superscripts 1–4 denote which PICO(s) the study contributed to. An asterisk (*) marks at least one KCS case with insufficient information on recovery. Doses and intervals are presented as reported in the source

First author	Country	Study design	Study population	Disease condition treated	Intervention (dose)	Comparator (dose)	Adverse effects (severe)	Adverse effects (mild)
Aguirre 1973^3^	USA	Case report	Dogs	NR	Sulfisoxazole with phenazopyridine HCL and Salicylazosulfapyridine	NA	NNS*	NR
Altreuther et al. 2011^2^	Germany, France, Portugal and Albania	Randomized controlled trial, multi center	Owned dogs (*n* = 26)	Isospora/nematode infection	Sulfadimethoxine (40 mg/kg q24h day 0, 25 mg/kg q24h thereafter)	NA	0	0
Anderson et al. 1984^3^	USA	Case report	Owned dog (*n* = 1)	Reccutrant E-coli Cystitis	Trimethoprim-Sulfadiazine	NA	1	0
Anonymous 1995^3^	Canada	Retrospective observational	Dogs and cats	UTI and NR	Sulfadizine	NA	1	3
Anyogu et al. 2018^3,4^	Nigeria	Randomized controlled trial, experimental, single center	Dogs (*n* = 5)	NA	Trimethoprim-Sulphamethoxazole (30, 60 or 120 mg/kg q12h)	NA	15	NR
Barber and Trees 1996^1,2,3^	UK	Observational, multi center	Owned dogs (*n* = 10)	N caninum	Trimethoprim-Sulfamethoxazole/Sulphadiazine (15–30 mg/kg q12h)	Clindamycin, Pyrimethamine	0	3
Bedford 1985^3^	UK	Case report	Owned dogs	Various	Sulphasalazine	NA	6*	0
Berger et al. 1995^2,3^	USA	Prospective observational, multi center	Owned dogs (*n* = 33)	Various	Trimethoprim-Sulfadiazine (8,6–104 mg/kg/day)	NA	5*	0
Bourdeau 1998^2^	France	Prospective observational	Owned dogs (*n* = 61)	Pyoderma	Baquiloprim- Sulphadimethoxine (average 30 mg/kg q48h)	NA	0	0
Brahmstaedt et al. 1983a^2,3^	Germany	Retrospective observational, single center	Owned dogs (*n* = 111) and cats (*n* = 11)	Various	Trimethoprim-Sulfamerazin (30–40 mg/kg q12)	NA	0	13
Brahmstaedt et al. 1983b^2^	Germany	Retrospective observational, single center	Owned dogs (*n* = 355) and cats (*n* = 244)	Various	Sulfamerazine (25–50 mg/kg)	NA	0	0
Brenner et al. 2009^3^	USA	Case report	Owned dog (*n* = 1)	LRT infection	Trimethoprim-Sulfamethoxazole (40 mg/kg q12h)	NA	1	0
Brunnthaler 1977^2^	Germany	Prospective observational, single center	Owned dogs (*n* = 45)	Coccidiosis	Sulfadimethoxin/Dulfaguandin/Sulfadiazin (30–200 mg/kg)	NR	NR	NR
Bryan 1943^2^	USA	Prospective observational	Owned dogs (*n* = 16) and cats (*n* = 4)	Distemper/dysenteri/diarrhea	Succunylsulfathiazole (0,25 − 0,5Gm/kg q4-6 h)	NA	0	0
Campbell 1983^1^	USA	Randomized controlled trial, multi center	Owned dogs (*n* = 23)	Tonsillitis	Trimethoprim-Sulfadiazin	Novobiocin Sodium, Tetracycline, Anacetin, Princillin, Amoxicillin	NR	NR
Cannon 1976^2,3^	USA	Prospective observational, single center	Owned dogs (*n* = 23) and cats (*n* = 28)	Alimentary, respiratory, wound- or urogenital bacterial infections	Trimethoprim-Sulfadiazine (approx. 30 mg/kg/day, one individual double dose)	NA	0	NNS
Chretin et al. 2007^2^	USA	Randomized controlled trial, multi center	Owned dogs (*n* = 36)	Lymphoma or Osteosarcoma	Trimethoprim-Sulfadiazine (20–30 mg/kg q12h)	NA	0	0
Clare et al. 2014^1,2^	USA	Randomized controlled trial, single center	Owned dogs (*n* = 20)	Urinary tract infection	Trimethoprim-Sulfamethoxazole (15 mg/kg q12h)	Cephalexin (20 mg/kg q12h)	0	0
Collin et al. 1986^3^	USA	Case report	Owned dog (*n* = 1)	Dysuria	Trimethoprim-Sulfamethoxazole (15 mg/kg q12h)	NA	0	1
Delmage and Payne-Johnson 1991^3^	UK	Case report	Owned dog (*n* = 1)	Otitis externa and pyoderma	Trimethoprim-Sulphamethoxazole (10 mg/kg q12h)	NA	1	0
Diehl and Roberts 1991^3^	USA	Retrospective observational, single center	Owned dogs (*n* = 16)	Various	Trimethoprim-Sulfamethoxazole/Sulfonamide/Sulfadiazine or Sulfasalazine (11,7–65,7 mg/kg q12h)	NA	14*	2
Dodds 1997^3^	USA	Case report	Owned dog (*n* = 1)	Prophylax	Trimethoprim-Sulfonamide	NA	1	0
Dunbar and Foreyt 1985^2^	USA	Prospective experimental	Laboratory dogs (*n* = 7)	Coccidiosis	Ormetoprim-Sulfadimethoxine (33–66 mg/kg/day)	NA	0	0
Durr 1976^2^	Germany	Retrospective observational, single center	Owned dogs (*n* = 100) and cats (*n* = 27)	Coccidiosis	Sulfadiazin (15–30 mg/kg q12h)	NA	NR	NR
Eads 1949^4^	USA	Prospective observational, single center	Owned dogs (*n* = 52) and cats (*n* = 2)	Various infections	Sulfamerazine (about 143 mg/kg/day divided in 3–4 doses)	NA	0	0
England et al. 2007^2^	UK	Prospective observational	Owned dogs (*n* = 22)	Pyometra	Potentiated sulphonamides	NA	0	0
Fernando 1956^2^	Sri Lanka	Prospective observational	Dogs (*n* = 14)	Coccidiosis	Sulphamezathine/Sulphaguanidine (147 mg/kg/day dividend in 2 doses day 1, thereafter 98 mg/kg/day divided in 2 doses)	NA	NR	NR
Fox et al. 1993^3^	USA	Retrospective observational, single center	Owned dogs (*n* = 2)	Pyoderma and Otitis Externa	Trimethoprim-Sulfadiazine (25–30 mg/kg q12h)	NA	2	0
Frank et al. 1989^3^	Sweden	Case report	Owned dog (*n* = 1)	Prostatitis	Trimethoprim-Sulfadiazine (32 mg/kg/day divided in 2 doses)	NA	0	1
Frank et al. 2005^2,3^	USA	Prospective experimental	Owned dogs (*n* = 6)	None	Trimethoprim-Sulfamethoxazole (14,1–16 mg/kg q12h)	NA	0	≥ 5
Funk-Keenan et al. 2012^3^	USA	Prospective observational, multi center	Owned dogs (*n* = 34)	NR	Trimethoprim-Sulfamethoxazole/Sulfadiazine or Ormetoprim-Sulfadimethoxine (25,4–68,4 mg/kg/day)	NA	18	0
Gehring et al. 1971^2,3^	Germany	Retrospective observational, multi center	Owned dogs (*n* = 152) and cats (*n* = 22)	Various	Trimethoprim-Sulfadoxin (15 mg/kg once or twice)	NA	0	1
Giger et al. 1985^3^	USA	Retrospective observational, single center	Owned dogs (*n* = 6)	Scrotal pyoderma	Trimethoprim-Sulfadiazine (1 dog: 27,4 mg/kg twice, rechallange 22,9 mg(S)/kg twice)	NA	6	0
Gookin et al. 1999^3^	USA	Case report	Owned dog (*n* = 1)	URTI or Coccidiosis	Trimethoprim-Sulfadiazine (24 mg/kg q12h)	NA	1	0
Gray 1990^3^	UK	Retrospective observational	Dogs (*n* = 13)	NR	Trimethoprim-Sulphonamide	NA	13	0
Grondalen 1987^3^	Norway	Retrospective observational	Owned dogs (*n* = 7)	NR	Trimethoprim-Sulphonamide	NA	7	0
Hall et al. 1993^2,3^	USA	Prospective observational	Owned dogs (*n* = 21)	Pustular dermatitis	Trimethoprim-Sulfamethoxazole (30 mg/kg q12h)	NA	0	≥ 13
Halman et al. 2024^3^	Australia	Case report	Owned cat (*n* = 1)	Intracranial nocardiosis	Trimethoprim-Sulfadocine/Sulfadiazine (25–32 mg/kg q12h)	NA	1	0
Hardie and Barsanti 1982^2^	USA	Retrospective observational, single center	Owned dogs (*n* = 12)	Canine Actinomycosis	Triple sulfonamides, Trimethoprim-Sulfonamide and/or Sulfadimethoxine (8–15 mg/kg BID (Triple sulfonamides), 3–7 mg/kg BID (trimethoprim in the trimethoprim-sumfonamide combinations))	Procaine penicillin, Chloramphenicol (5 400 − 16 000IU/kg q12h)	0	0
Harvey 1987^3^	NR	Retrospective observational	Owned dogs (*n* = 2)	Superficial Pyoderma	Trimethoprim-Sulphadiazine (30 mg/kg q12h)	NA	2	0
Itoh and Muroaka 2002^2^	Japan	Prospective observational	Dogs (*n* = 34)	Isospora infection	Sulfadimethoxine/Sulfamonmethoxine (50 mg/kg q24h)	NA	0	0
Jeong et al. 2023^3^	Korea	Case report	Owned dog (*n* = 1)	Soft stools	Trimethoprim-Sulfamethoxazole (15 mg/kg q12h)	NA	1	0
Johnson et al. 2023^3^	USA	Retrospective observational, single center	Owned dogs (*n* = 2)	Pneumocytosis	Trimethoprim-Sulfamethoxazole (15–30 mg/kg q12h)	NA	2	0
Kose et al. 2021^2^	Turkey	Prospective observational, single center	Shelter dogs (*n* = 22)	Respiratory tract infections	Trimethoprim-Sulfamethoxazole (25 mg/kg q24h)	Enrofloxacin, Chloramphenicol, Amoxicillin-Clavulanic acid, Erythromycin (8,75-50 mg/kg q12-24 h)	NR	NR
Kunkle et al. 1995^1,2,3^	USA	Prospective observational, single center	Owned dogs (*n* = 196) and cats (*n* = 24)	Various	Trimethorprim-Sulfadiazine	Amoxicillin, Amoxicillin-Clavulanic acid, Ampicillin, Cefadroxil, Cephalexin, Cephradine, Chloramphenicol, Enrofloxacin, Erythromycin stearate, Lincomycin, Tetracycline	0	42
Lavergne et al. 2008^3^	UK	Retrospective case control	Owned dogs (*n* = 45)	NR	Trimethoprim-Sulphamethoxazole/Sulphadiazine or Ormetoprim-Sulphadimethoxine	NA	34	0
Lees et al. 1986^3^	USA	Case Report	Dog (*n* = 1)	Pyoderma	Trimethoprim-Sulfadiazine (36 mg/kg q12h)	NA	1	0
Lefkaditis 2005^2^	Greece	Prospective observational	Dogs (*n* = 30)	Pyodemodecosis	Trimethoprim-Sulfadiazine (30 mg/kg q12h)	NA	NR	NR
Lewis et al. 2023^3^	USA	Retrospective observational	Dog (*n* = 1)	Dermatology	Trimethoprim-Sulfamethoxazole (30 mg/kg q12h)	NA	1	0
Ling et al. 1984^1^	USA	Prospective observational, single center	Owned dogs (*n* = 204)	UTI	Trimethoprim-Sulfadiazine/Sulfamethoxazole (approx. 25 mg/kg q12h)	Ampicillin (77–110 mg/kg q8h)	NR	NR
Ling and Ruby 1979^2^	USA	Prospective observational, single center	Owned dogs (*n* = 84)	UTI	Trimethoprim-Sulfadiazine/Sulfamethoxazole (26,4 mg/kg/day divided in two doses)	NA	NR	NR
Little 1990^3^	UK	Case report	Dog (*n* = 1)	Pyoderma	Trimethoprim-Sulphadiazine	NA	1	0
Marino and Jaggy 1993^3^	USA	Case report	Owned dog (*n* = 1)	Nocardiosis	Trimethoprim-Sulfamethoxazole (15–40 mg/kg)	NA	1	0
McCandlish and Thompson 1979^2^	UK	Randomized controlled trial, experimental	Laboratory dogs (*n* = 6)	Bordetellosis	Trimethoprim-Sulphadiazine (24 per cent suspension at a dose rate of 1 ml/8,2 kg body weight q24h)	NA	0	0
McEwan 1992^3^	Scottland	Case report	Owned dog (*n* = 1)	Generalised demodectic mange and secondary deep pyoderma	Trimethoprim-Sulphamethoxazole (28 mg/kg)	NA	1	0
Medleau et al. 1990^3^	USA	Retrospective observational	Owned dogs (*n* = 6)	Different skin problems, Hemorrhagic diarrhea	Trimethoprim-Sulfamethoxazole/Sulfadiazine (12–32 mg/kg q12-24 h)	NA	6	0
Messinger and Beale 1993^2,3^	USA	Randomized controlled trial, single center	Owned dogs (*n* = 45)	Pyoderma	Trimethoprim-Sulfadiazine or Ormethoprim-Sulfadimethoxine (27,5–80 mg/kg q12-24 h)	NA	2	1
Morgan and Bachrach 1982^3^	USA	Retrospective observational, single center	Owned dogs (*n* = 14)	Various	Sulfasalazine or Trimethoprim-Sulfadiazine (12,5–100 mg/kg q8h)	NA	7	7
Nak et al. 2009^2^	Turkey	Prospective observational, single center	Owned cats (*n* = 10)	Pyometra	Trimethoprim-Sulphadoxine (15 mg/kg q24h)	NA	0	0
Noli et al. 1995^2,3^	The Netherlands	Retrospective observational, single center	Owned dogs and cats (total *n* = 2000)	Varied	Trimethoprim-Sulphadiazine/Sulpfatroxazole/Sulphmethoxazole/Succinylsulphathiazole	NA	21	0
Nuttall and Malham 2004^3^	UK	Case report	Owned dog (*n* = 1)	Wound infection	Trimethoprim-Sulphadiazine	NA	1	0
Osweiler and Green 1978^3^	USA	Pro- and retrospective observational experimental, single center	Kennel and laboratory dogs (*n* = 13)	Coccidiosis	Sulfaquinoxaline	NA	13	0
Patra and Tripathy 1986^2^	India	Prospective observational, single center	Dogs (*n* = 32)	GI disaese	Trimethoprim-Sulphadiazine (30 mg/kg/day divided in two doses)	NA	NR	NR
Patterson and Grenn 1975^3^	Canada	Retrospective observational, single center	Kennel dogs (*n* = 12)	Coccidiosis	Sulfaquinoxaline	NA	12	0
Pena-Ramos et al. 2021^3^	UK	Case report	Owned dog (*n* = 1)	Colitis	Sulfasalazine (15 mg/kg q12h)	NA	1	0
Rogers et al. 1988^1^	USA	Randomized controlled trial, experimental, single center	Laboratory dogs (*n* = 12)	UTI	Trimethoprim-Sulfadiazine (30 mg/kg once or 15 mg/kg q12h)	Amikacin (10 mg/kg q12h or 20 mg/kg once)	NR	NR
Rowland et al. 1992^3^	USA	Case report	Owned dog (*n* = 1)	Pyoderma	Trimethoprim-Sulfadiazine (30 mg/kg q12h)	NA	1	0
Rubin et al. 1998^3^	Canada	Case report	Owned dog (*n* = 1)	UTI	Trimethoprim-Sulfamethoxazole (33 mg/kg q12h)	NA	1	0
Samuel et al. 1990^2^	India	Prospective observational, single center	Dogs (*n* = 14)	Demodicosis	Trimethoprim-Sulphamethoxasole (480 mg q12h)	NA	NR	NR
Sansom et al. 1985^3^	UK	Retrospective observational	Owned dogs (*n* = 13)	Colitis	Sulphasalazine (250–500 mg q12-24 h)	NA	12	1
Sansom and Barnett 1985^3^	UK	Retrospective observational, single center	Owned dogs	Colitis	Salicylazosulphapyridine	NA	3*	0
Scott and Miller 1999^3^	USA	Retrospective observational, single center	Owned dogs (*n* = 8)	Various	Ormetoprim-Sulfadimethoxine or Trimethoprim+Sulfamethoxzole/Sulfadiazin	NA	8	0
Scott et al. 1993^,2,3^	USA	Prospective observational, single center	Owned dogs (*n* = 20)	Staphylococcal pyoderma	Ormetoprim-Sulfadimethoxine (27,5–55 mg/kg q24h)	NA	0	1
Scott et al. 1986^3^	USA	Case report	Owned dog (*n* = 1)	Urinary tract infection	Trimethoprim-Sulfamethoxazole	NA	1	0
Seelig et al. 2008^3^	USA	Case report	Owned dog (*n* = 1)	Aspiration Pneumonia	Trimethoprim-Sulfamethoxazole (30 mg/kg q12h)	NA	1	0
Seemanthini and Vinodkumar 2016^2^	India	Prospective observational, single center	Shelter dogs (*n* = 12)	Isospora/diarrhoea	Trimethoprim-Sulphonamide (15 mg/kg)	NA	0	0
Seena et al. 2005^1,2,4^	India	Randomized controlled trial	Owned dogs (*n* = 6)	Pyoderma	Trimethoprim-Sulfamethoxazole (30 mg/kg q12h)	Lincomycin, Cephalexin (22–25 mg/kg q12h)	NR	NR
Slatter and Blogg 1978^3^	Australia	Retrospective observational, single center	Dogs (*n* = 14)	NR	Sulphadiazine, Salicylazosulphapyridine	NA	1	13
Sullivan et al. 1992^3^	USA	Case report	Owned dog (*n* = 1)	Vaginitis	Trimethoprim-Sulfadiazine (18,8 mg/kg q12h)	NA	1	0
Sutton and Roach 1988^3^	UK	Case report	Owned dog (*n* = 1)	Kennel cough	Trimethoprim-Sulphadiazine (15 mg/kg q12h)	NA	0	1
Taeymans and O’Marra 2009^3^	USA	Case report	Owned dog (*n* = 1)	Neospora and Weakness	Trimethoprim-Sulfamethoxazole	NA	1	0
Taksdal 1987^3^	Norway	Retrospective observational, multi center	Owned dogs (*n* = 4)	Various	Trimethoprim+Sulfadiazin	NA	4	0
Tarlow et al. 2005^2,3^	USA	Retrospective observational, single center	Owned dogs (*n* = 4)	Neurologic toxoplasmosis	Trimethoprim-Sulfamethoxazole (15 mg/kg q12h)	NA	2*	0
Tham et al. 2016^3^	USA	Case report	Owned dog (*n* = 1)	C. bigenetica cutaneous infection	Trimethoprim-Sulfamethoxazole (15–23 mg/kg q12-24 h)	NA	1	0
Thomson 1990^3^	Canada	Case report	Owned dog (*n* = 1)	Suspected endometritis	Trimethoprim-Sulfamethoxazole (20,9 mg/kg q12h)	NA	1	0
Thornburg 1988^3^	USA	Retrospective observational	Dogs	Pyoderma	Trimethoprim-Sulfadiazine	NA	2	0
Thrusfield et al. 1991^2^	UK	Retrospective observational, multi center	Owned dogs (*n* = 190)	Kennel cough	Trimethoprim-Sulphonamide	Oxytetracycline, Ampicillin, Amoxycillin	NR	NR
Tjalve 1997^3^	Sweden	Retrospective observational, multi center	Dogs (*n* = 3)	NR	NR	NA	3*	0
Tjalve et al. 2018^3^	Sweden	Retrospective observational	Owned dog (*n* = 1)	Suspected pyometra	Trimethoprim-Sulphametoxazole	NA	0	1
Tjalve et al. 2017^3^	Sweden	Retrospective observational, multi center	Dog (*n* = 1)	NR	Trimethoprim-Sulphametoxazole	NA	0	1
Tjälve 1997^3^	Sweden	Retrospective observational, multi center	Dogs (*n* = 17) and cats (*n* = 2)	NR	Sulfadiazine, Sulfadoxin or Sulfamethoxazol	Cephalexin, Enrofloxacin, Erythromycin, Phenoxymethylpenicillin, Klindamycin, Ampicillin, Metronidazole, Doxycycline, Amoxicillin-Clavulanic acid, Benzylpencillin, Kloramfenikol, Lincomycin, Tetracyklin, Procaine benzylpenicillin, Pivampicillin, Cefachlor	8*	11
Tjalve et al. 2019^3^	Sweden	Retrospective observational, multi center	Dogs	Prostatitis	Trimetoprim-Sulfametoxazol/Sulfadiazin	NA	3*	0
Todenhofer 1969^3^	Germany	Retrospective observational, single center	Owned dogs (*n* = 8)	Profylaxis	Sulfadiazin	NA	2	6
Torres et al. 1996^3^	USA	Case report	Owned dog (*n* = 1)	Pneumonia	Trimethoprim-Sulphadiazine (25 mg/kg q12h)	NA	1	0
Trapp et al. 2005^3^	Brazil	Case report	Owned dog (*n* = 1)	NR	Trimethoprim-Sulfadiazine	NA	1	0
Trepanier 2015^3^	USA	Case report	Owned dog (*n* = 1)	Stranguria and Pollakiuria	Trimethoprim-Sulfamethoxazole (15 mg/kg q12h)	NA	1	0
Trepanier et al. 2003^3^	USA	Retrospective observational, multi center	Owned dogs (*n* = 40)	Pyoderma, otitis, wounds, lower urinary tract signs, prophylaxis, tooth abscess and coccidia	Trimethoprim-Sulfamethoxazole/Sulfadiazine or Ormetoprim-Sulfadimethoxine (23,4–81,4 mg/kg/day)	NA	40	0
Trimborn and Vick 1992^3^	Germany	Case report	Owned dog (*n* = 1)	Pyodermia	Sulfamethoxazole (25–50 mg/kg q12-24 h)	NA	1	0
Tripathy 1990^2^	India	Prospective observational	Owned dogs (*n* = 10)	Demodex infection	Trimethoprim-Sulphadiazine (15 mg/kg q12h)	NA	0	0
Tuntivanich et al. 1997^3^	Thailand	Case report	Dog (*n* = 1)	Diarrhoea	Trimethoprim-Sulfadimethylpyrimidine	NA	0	1
Turnwald et al. 1986^2^	USA	Prospective observational, experimental	Laboratory dogs (*n* = 18)	Cystitis	Trimethoprim-Sulfadiazine (15 mg/kg q12h or 60–90 mg/kg once)	NA	NR	NR
Twedt et al. 1997^3^	USA	Retrospective observational	Owned dogs (*n* = 4)	Tracheobronchitis, pyoderma, UTI	Trimethoprim-Sulfadiazine/Sulfamethoxazole (18–53 mg/kg q12h)	NA	4	0
Vasilopulos et al. 2005^1^	USA	Case report	Owned dog (*n* = 1)	Dermatitis	Ormetoprim-Sulfadimethoxine 30–60 mg/kg q24h	NA	1	0
Weiss and Adams 1987^3^	USA	Case report	Owned dog (*n* = 1)	Paragonimiasis with bronchitis and bronchiectasis. Possible sec. Bacterial pneumonia	Trimethoprim-Sulfadiazine (15 mg/kg q12h)	NA	1	0
Weiss and Klausner 1990^3^	USA	Case report	Owned dog (*n* = 1)	Paragonimus infection and possible sec. Bacterial pneumonia	Trimethoprim-Sulfadiazine (14 mg/kg q12h)	NA	1	0
Werner and Bright 1983^3^	USA	Retrospective observational, single center	Owned dogs (*n* = 2)	Pyoderma	Trimethoprim-Sulfadiazine	NA	2	0
Whur 1987^3^	UK	Case report	Owned dog (*n* = 1)	Pyoderma	Trimethoprim-Sulphamethoxazole (10 mg/kg q8h)	NA	1	0
Wilkinson 1977^2,3^	Australia	Retrospective observational, single center	Laboratory cats (*n* = 63)	Isosporosis	Sulphadimethoxine, Sulphadimidine (50 mg/kg/day)	NA	0	NNS
Williamson et al. 2002^2,3^	USA	Prospective observational	Owned dogs (*n* = 7)	None	Trimethoprim-Sulfamethoxazole (26,5–31,3 mg/kg q12h)	NA	6	0
Younas et al. 2014^1,2^	Pakistan	Prospective observational	Dogs (*n* = 15)	Coccidiosis	Sulfadimethoxine (60 mg/day)	Furazolidone (20 mg/kg/day)	NR	NR

^1^ PICO 1, ^2^ PICO 2, ^3^ PICO 3, ^4^ PICO 4, NR Not reported, NA Not applicable, NNS Number not specified, * At least one patient with KCS without information about recovery

*TMS* potentiated sulfonamides, *AE* adverse event, *KCS* keratoconjunctivitis sicca, *NR* not reported, *NA* not applicable, *NNS* number not specified, q8h/q12h/q24h, every 8/12/24 hours; SID/BID/TID, once/twice/three times daily

### PICO 1

Are adverse events more common in cats and dogs treated with TMS in comparison to other antimicrobials?

### Included studies

There were eight studies that included a comparison between TMS treatment (*n* = 666) and other antimicrobial substances (*n* = 875) in dogs (Campbell 1983; Ling et al. 1984; Rogers et al. 1988; Thrusfield et al. 1991; Kunkle et al. 1995; Seena et al. 2005; Clare et al. 2014; Younas et al. 2014). Four studies were RCTs (Campbell 1983; Rogers et al. 1988; Seena et al. 2005; Clare et al. 2014) and four studies were observational cohorts (Ling and Ruby 1979; Thrusfield et al. 1991; Kunkle et al. 1995; Younas et al. 2014). Further study characteristics are reported in Table 5. Only one study (Kunkle et al. 1995) investigated AE in cats treated with TMS (*n* = 24) and other antimicrobials (*n* = 125).

### Effect size

**Fig. 3:**
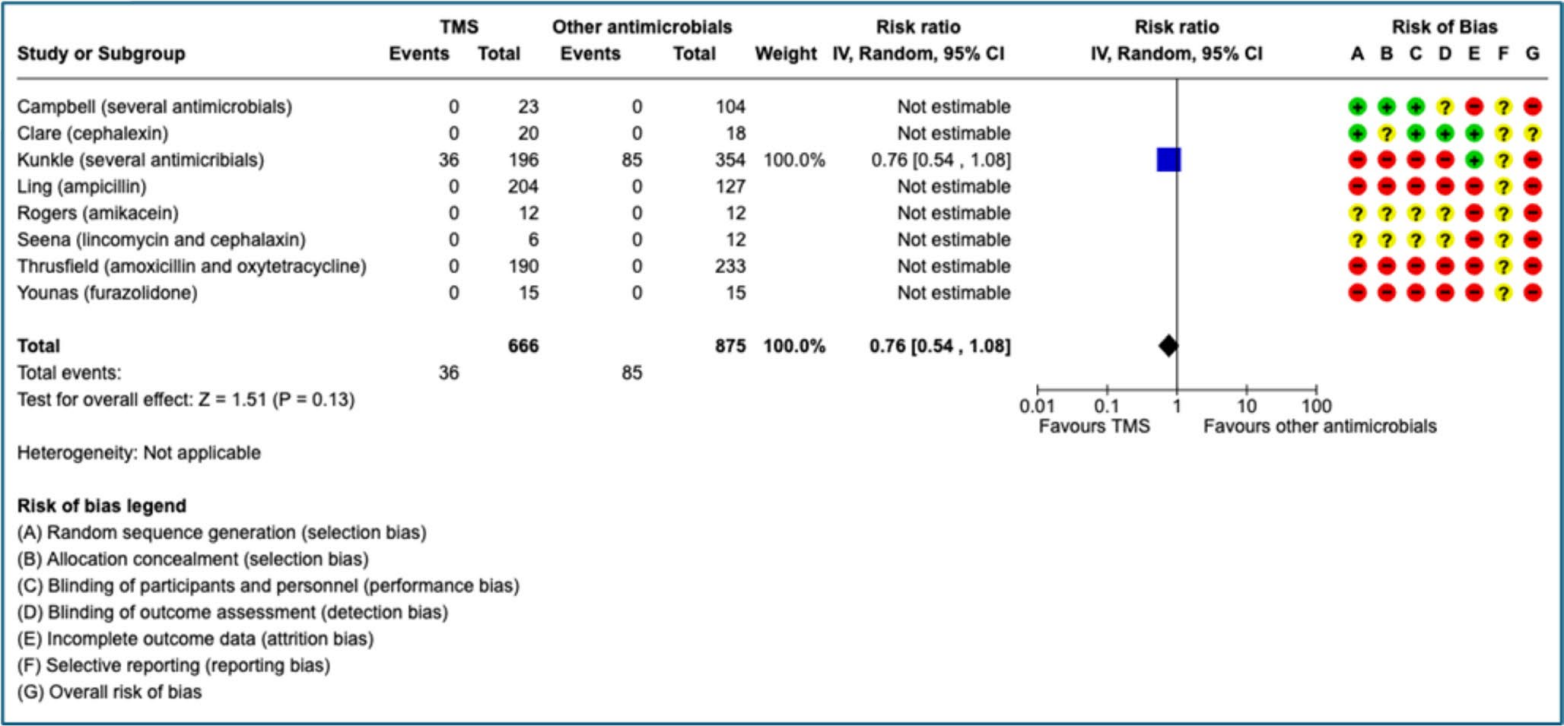
Relative effects size of adverse events and 95% confidence interval (CI) shown in a pairwise comparison of dogs treated with trimetoprim sulphonamide (TMS) or other antimicrobials. Other antimicrobial substances are shown in brackets after the study author name. In the risk of bias assessment a red “traffic light” mean high risk of bias, yellow means that there are some concerns and green that the risk of bias is low. The different domains are reported in the figure. Forest plot created with the RevMan web software (revman.cochrane.org)

There were more AE in dogs treated with other antimicrobials in comparison to TMS and AE only occurred in one of the trials (Kunkle et al. 1995). Relative effect (risk ratio) size for dogs is reported in the forest plot (Fig. 3). The relative effect did not change when studies with zero events were excluded from the analysis. The absolute risk difference with TMS was 23 fewer AE (from 45 fewer to 8 more, 95% CI) per 1000 dogs in comparison to other antimicrobials. All reported AE were mild and no immune mediated conditions were reported. Conversely, there were more AE in cats that were treated with TMS in comparison to other antimicrobials. Relative effect was 1.16 (0.54–2.5) for cats, and the absolute risk difference for cats was 35 more AEs per 1,000 (95% CI 99 fewer to 324 more) with TMS versus comparators.

### Risk of bias

The overall risk of bias was high across studies since few studies specifically reported AE. The present authors made the assumption that severe AE were likely to be reported in most studies, but mild events are likely to be underreported in several studies and confer a high risk of attrition bias (incomplete reporting). There were four observational studies (Ling et al. 1984, Thrusfield et al. 1991; Kunkle et al. 1995; Younas et al. 2014) with a high risk of selection bias (randomization and allocation) but this is unlikely to be as relevant in the reporting of AE as with treatment efficacy since the present PICO includes all cats and dogs and not only animals with specific conditions. Reporting bias was uncertain in all studies due to no predefined protocols. All domains in the risk of bias assessment for all dog studies are reported in Fig. 3.

### Certainty of evidence

The certainty of evidence starts out as high if the evidence comes from randomized trials and low if it comes from non-randomized trials (Guyatt et al. 2025). Half of the included trials were non-randomized, but since there was minor incongruence between study designs and randomization is unlikely to have a major effect on AE (in terms of treatment efficacy it would have been different) it was decided that evidence started out as high. The overall risk of bias was high, especially in regard to incomplete reporting of outcomes, and the body of evidence was downgraded two levels from high to low. There were no concerns of indirectness, inconsistency, imprecision or dissemination bias for dogs. The overall certainty for cats started out as low as there were no randomized studies and it was further downgraded for risk of bias and imprecision because the 95% CI crossed three of the clinical thresholds (small, moderate and large thresholds). The overall certainty of evidence for cats was very low. All domains and overall certainty can be seen in Summary of Findings (SoF) tables in supplementary Table 1.

#### PICO 2

What proportion of TMS treatments in dogs and cats result in adverse events?

There were 31 studies reporting TMS treatment for several different disease conditions in 3776 dogs (Eads 1949; Fernando 1956; Gehring et al. 1971; Cannon 1976; Durr 1976; Brunnthaler 1977; Ling and Ruby 1979; Hardie and Barsanti 1982; Brahmstaedt et al. 1983a; Brahmstaedt et al. 1983b; Patra and Tripathy 1986; Samuel et al. 1990; Tripathy 1990; Thrusfield et al. 1991; Hall et al. 1993; Messinger and Beale 1993; Scott et al. 1993; Berger et al. 1995; Kunkle et al. 1995; Noli et al. 1995; Barber and Trees 1996; Bourdeau 1998; Itoh and Muroaka 2002; Lefkaditis 2005; Chretin et al. 2007; England et al. 2007; Altreuther et al. 2011; Clare et al. 2014; Younas et al. 2014; Seemanthini and Vinodkumar 2016; Kose et al. 2021). Study characteristics including dosage and disease conditions are reported in Table 5. Eight studies reported TMS treatment in 429 cats but only one study (Kunkle et al. 1995) reported the number of AE (*n* = 6). Two studies (Cannon 1976; Wilkinson 1977) reported that “some cats” and “a number of cats” had AE so it was not possible to determine what the pooled proportion was across studies. The rest of the studies (Gehring et al. 1971; Durr 1976, Brahmstaedt et al. 1983a; Brahmstaedt et al. 1983b; Nak et al. 2009) did not report any AE in cats so there were six known mild AE (and “some more”) in a population of 429 cats.

### Prevalance

**Fig. 4:**
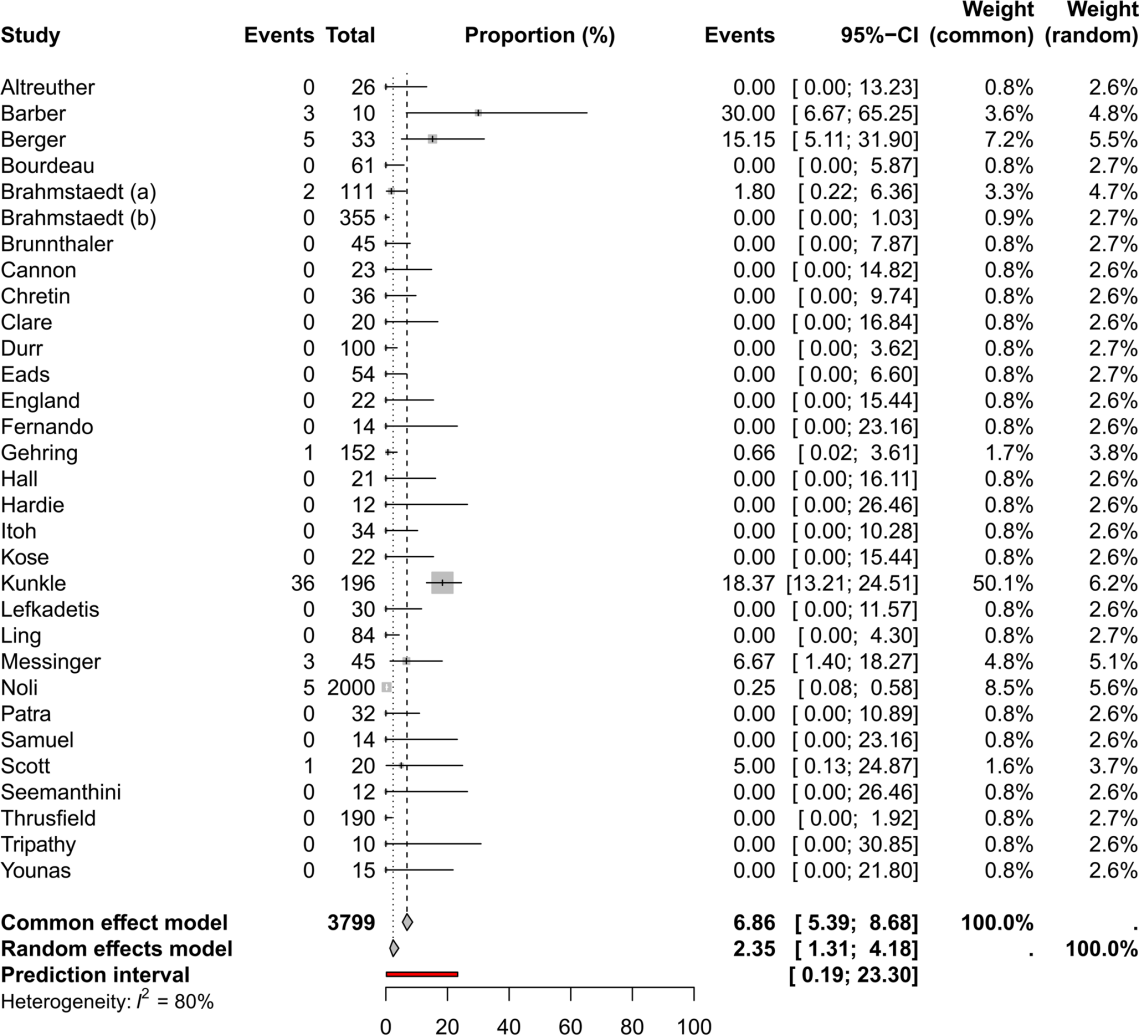
Forest plot of dogs treated with TMS showing number of adverse events, the total population and proportions, individual study weight expressed in both fixed and random effects

The pooled prevalence of all AE (*n* = 56) was 2.3% (CI 1.3–4.2%) in 3776 dogs that were treated with TMS. The number of events, weight and CI per each study is reported in Fig. 4.

### Severity of reported adverse events

Most of the events (36/56) were mild as reported by Kunkle et al. (1995) (see PICO 1). KCS was reported in 5 dogs out of which two dogs did not respond to ciclosporin treatment and could be considered severe (Berger et al. 1995). Noli et al. (1995) reported that AE occurred in 5/2000 (0.25%) dogs treated with TMS by their dermatological service. The severity was not described in detail but the prevalence was reported in the discussion section of a case series of severe AE so it is likely that these cases were severe AE rather than mild (Noli et al. 1995). A dog in another study was reported to have hepatotoxic AE (Messinger and Beale 1993), but it was not reported if the dog had hepatic necrosis or just a transient increase in hepatic enzymes. Other reported AE were gastrointestinal disease (*n* = 3), hematuria (*n* = 2), one seizure (*n* = 1) and personality change (*n* = 1). Mortality was not reported in any of the studies. In summary, the total number of severe events in a population of 3776 is likely to be less than 10 in the whole population, which is approximately 0.2%.

Number of AE, proportions, weight and confidence intervals and random and fixed effect for the proportional meta-analysis for PICO 2 can are shown in supplementary Table 2.

### Certainty of evidence

There is no formal GRADE guidance to assess certainty of evidence in proportional meta-analysis but the same domains have here been applied as in a pairwise comparison. Risk of bias was high across studies due to the same reasons as for PICO 1 where mild AE are likely to be underreported. The body of evidence was thus downgraded one level from low (most are observational studies which meansthat the certainty of evidence starts out as low). I2 (the percentage of variability attributableto between-study heterogeneity rather than chance) was high (80%), but this is likely explained by incomplete reporting, which we had already accounted for; therefore, we did not downgrade for inconsistency.The 95% CI did not cross any clinical thresholds (small effect threshold was 10%) for either random or fixed effect so there were no concerns with imprecision. The overall certainty of evidence was very low.

#### PICO 3

What adverse events have been reported in TMS treatment of cats and dogs?

Eighty studies described AE in cats and dogs. Keratoconjunctivitis sicca was reported in 23 studies in 109 dogs.Severe reactions were documented in 64 studies and in 245 dogs and 2 cats. Mild events were documented in 23 studies and at least 92 dogs and 15 cats (both dogs and cats were reported in groups without further specifications in some studies).

### Keratoconjunctivitis sicca

Keratoconjunctivitis sicca (KCS) was reported in 109 dogs and 23 publications (Todenhofer 1969; Aguirre 1973; Slatter and Blogg 1978; Morgan and Bachrach 1982; Bedford 1985; Sansom and Barnett 1985; Sansom et al. 1985; Collin et al. 1986; Sutton and Roach 1988; Diehl and Roberts 1991; Marino and Jaggy 1993; Berger et al. 1995; Tjalve 1997; Tjälve 1997; Tuntivanich et al. 1997; Twedt et al. 1997; Trepanier et al. 2003; Nuttall and Malham 2004; Frank et al. 2005; Tarlow et al. 2005; Trapp et al. 2005; Lavergne et al. 2008; Tjalve et al. 2019), but no reports on KCS in cats were identified. Reported KCS cases were from 33 different breeds (Supplementary Table 3), and most commonly reported in German shepherds (9), Cocker spaniels (9) and Dachshunds (6). Most used TMS were sulfadiazine (SDZ) + trimethoprim (TMP) (38 reports), sulfasalazine (SZZ) (16 reports) and sulfamethoxazole (SMX) + TMP (12 reports) (Supplementary Table 3). The doses used were 8,6–104 mg/kg when dosed once daily and 11–65,7 mg/kg when administered twice daily. The onset of KCS was reported to occur between 18 h and 2 years after treatment was initialized. Five studies reported that the clinical signs of the AE started after a treatment duration up to a week (Aguirre 1973; Diehl and Roberts 1991; Berger et al. 1995; Tuntivanich et al. 1997; Trepanier et al. 2003). In one of those it is not clear in how many dogs KCS was diagnosed within a week of TMS therapy because only the range 5–36 days and mean 12 days were reported (Trepanier et al. 2003).

### Polyarthritis (arthropathy)

Polyarthritis (PA) was reported in 45 dogs in 14 studies (Werner and Bright 1983; Giger et al. 1985; Lees et al. 1986; Grondalen 1987; Harvey 1987; Taksdal 1987; Whur 1987; Gray 1990; Little and Carmichael 1990; Medleau et al. 1990; Noli et al. 1995; Trepanier et al. 2003; Lavergne et al. 2008; Funk-Keenan et al. 2012), but no reports on PA in cats were identified. In 22 of these cases, the dogs were reported to recover after discontinuation of TMS. Information about whether this was due to withdrawal of TMS of medication against PA was not extracted in this review. In the remaining 23 dogs, no information about recovery were reported. Polyarthritis was reported in 17 breeds (supplementary Table 3) with most reports in Doberman pinchers (10 reports), Golden retrievers (4 reports) and Gordon setters (4 reports). Most commonly, sulfadiazine (9 reports) or sulfamethoxazole (3 reports) combined with trimethoprim was used before onset of PA (supplementary Table 3). The clinical signs of PA started < 1 to 36 days after drug was started. In seven studies the clinical signs started ≤ 7 days after initialization of TMS treatment (Werner and Bright 1983; Lees et al. 1986; Whur 1987; Little and Carmichael 1990; Medleau et al. 1990; Noli et al. 1995; Trepanier et al. 2003), with one study reporting a mean of 12 days and a range of 5–36 days for several AEs with no specification for PA (Trepanier et al. 2003). Three studies did not report when the clinical signs of the AE became detectable (Grondalen 1987; Lavergne et al. 2008; Funk-Keenan et al. 2012). In the remaining studies, the clinical signs of PA were reported to start after 7–21 days of TMS administration. The doses reported ranged between 23 and 81 mg/kg per day.

### Hepatic necrosis

Hepatic necrosis (HN) was reported in 28 dogs in 17 studies (Anderson et al. 1984; Thornburg 1988; Thomson 1990; Diehl and Roberts 1991; Rowland et al. 1992; Messinger and Beale 1993; Anonymous 1995; Noli et al. 1995; Dodds 1997; Tjalve 1997; Twedt et al. 1997; Trepanier et al. 2003; Lavergne et al. 2008; Funk-Keenan et al. 2012; Tjalve et al. 2018; Pena-Ramos et al. 2021; Johnson et al. 2023), no reports of HN in cats were identified. Four of the dogs with HN recovered, 13 were reported dead because of HN and no information on recovery was given in the remaining dogs. Hepatic necrosis was most commonly reported in Irish setters (3 dogs), Samoyeds (2 dogs) and Schnauzers (2 dogs). Other breeds are shown in supplementary Table 3. The sulfonamides used combined with trimethoprim were sulfamethoxazole (10 dogs), sulfadiazine (8 dogs) and sulfatroxazole (one dog). In the remaining dogs, the substances were not specified. The clinical signs of HN were reported to have started after more than 7 up to 30 days of treatment in most studies, with some studies reporting onset after the first dose (Messinger and Beale 1993), after 3 days (Twedt et al. 1997), after 5 days (Anonymous 1995) and after 7 days (Rowland et al. 1992; Tjalve et al. 2019). Doses were only reported in six studies and ranged from 15 mg/kg twice daily to 53 mg/kg twice daily (Thomson 1990; Diehl and Roberts 1991; Rowland et al. 1992; Messinger and Beale 1993; Twedt et al. 1997; Johnson et al. 2023).

### Severe hematological adverse events

Sixteen studies reported various severe hematological adverse events in dogs (Patterson and Grenn 1975; Osweiler and Green 1978; Weiss and Adams 1987; Weiss and Klausner 1990; McEwan 1992; Sullivan et al. 1992; Trimborn and Vick 1992; Fox et al. 1993; Noli et al. 1995; Trepanier et al. 2003; Tarlow et al. 2005; Funk-Keenan et al. 2012; Tham et al. 2016; Anyogu et al. 2018; Pena-Ramos et al. 2021; Jeong et al. 2023), in dogs from different breeds (Supplementary Table 3), but not reports on cats were identified. Four studies reported immune mediated thrombocytopenia (ITP) in 14 dogs (Osweiler and Green 1978; Trimborn and Vick 1992; Pena-Ramos et al. 2021; Jeong et al. 2023). Six of those dogs recovered and eight dogs died. The remaining studies reported of anemia (including e.g. aplastic anemia, immune mediated hemolytic anemia [IMHA]) in eight dogs (three dogs recovered, two dogs died and no information about recovery reported in three dogs) (Weiss and Adams 1987; Weiss and Klausner 1990; Fox et al. 1993; Funk-Keenan et al. 2012; Tham et al. 2016), non-regenerative anemia in one dog that recovered (Tarlow et al. 2005). and one study reported on hemorrhage in the small bowel and vulva in 12 miniature poodles from which six dogs recovered and six dogs died (Patterson and Grenn 1975). To 25 dogs suffering from ITP and hemorrhages, sulfaquinoxaline was administered with drinking water in doses not reported (Patterson and Grenn 1975; Osweiler and Green 1978). In four studies sulfamethoxazole-trimethoprim was administered in the doses 15–25 mg/kg twice daily and in one study sulfadiazine-trimethoprim was administered at 14–25 mg/kg twice daily (Weiss and Adams 1987; Weiss and Klausner 1990; Trimborn and Vick 1992; Fox et al. 1993; Tarlow et al. 2005; Funk-Keenan et al. 2012; Tham et al. 2016; Pena-Ramos et al. 2021; Jeong et al. 2023). The clinical signs of AE started between 1 and 365 days of treatment. In one study, reporting on hemolytic anemia in three dogs, neither TMS type or doses were reported (Funk-Keenan et al. 2012).

### Mild hematological adverse events

Three studies in dogs from different breeds reported various non-serious hematological AE from TMS (Williamson et al. 2002; Trepanier et al. 2003; Lavergne et al. 2008), but no reports on cats were identified. The reported observations were thrombocytopenia and neutropenia without clinical signs of AE.

*Dermatologic reactions and other immune mediated events* Seventeen studies reported various other immune-mediated reactions from TMS (Werner and Bright 1983; Scott et al. 1986; Medleau et al. 1990; Delmage and Payne-Johnson 1991; Marino and Jaggy 1993; Kunkle et al. 1995; Noli et al. 1995; Tjalve 1997; Tjälve 1997; Scott and Miller 1999; Trepanier et al. 2003; Nuttall and Malham 2004; Trapp et al. 2005; Lavergne et al. 2008; Funk-Keenan et al. 2012; Trepenier, 2015; Tjalve et al. 2017; Tjalve et al. 2018; Kose et al. 2021) in dogs from different breeds and in two cats (supplementary Table 3),. Those AE were various in both character and severity e.g. erythema multiforme, pemphigus foliaceus, skin rashes, urticaria, skin eruptions and ulcerations, fever, epidermal necrolyses, vasculitis, eye irritation, pustular dermatitis, “idiosyncratic sulfonamide toxicity” (i.e. stranguria, pollakiuria, ventral oedema, depression, increased alanine aminotransferase and neutropenia) and facial swelling. In cats, ulcerative dermatitis and ulcerations of oral mucus membranes were described. The onset of AE clinical signs were usually between 7 and 30 days, ranging from hours to 61 days after initialization of TMS therapy.

### Remaining conditions

Several different AE have been associated with either SDZ, SMZ or SDX combined with TMP and with sulfadimethoxine combined with ormetoprim. Doses used were 27.5 mg/kg once daily to 40 mg/kg twice daily and the clinical signs of the adverse effects started after 1 to 45 days of treatment. Gastro-intestinal AE (vomiting, diarrhoea, anorexia/loss of appetite etc.) has been reported in 64 dogs and 12 cats (Wilkinson 1977; Brahmstaedt et al. 1983a; Anderson et al. 1984; Marino and Jaggy 1993; Anonymous 1995; Kunkle et al. 1995; Barber and Trees 1996; Tjälve 1997; Trapp et al. 2005). In addition, salivation during drug administration has been reported on group level in cats (Cannon 1976; Brahmstaedt et al. 1983a). Adverse events on the thyroid gland (goitres hypothyroidism, iatrogenic hypothyroid crisis, or low levels of thyroid hormones) were reported in 23 dogs and in 1 cat (Hall et al. 1993; Torres et al. 1996; Gookin et al. 1999; Williamson et al. 2002; Frank et al. 2005; Seelig et al. 2008; Taeymans and O’Marra 2009; Brenner et al. 2009; Halman et al. 2024). Follow-up on thyroid recovery was limited and heterogeneous, so no typical time to normalization can be inferred. Adverse events from the urogenital tract (polydipsia, polyuria, haematuria and urolithiasis) have also been reported in both dogs and cats (Frank et al. 1989; Messinger and Beale 1993; Nuttall and Malham 2004; Vasilopulos et al. 2005; Lavergne et al. 2008; Tjalve et al. 2019; Lewis et al. 2023). Finally, conditions such as alopecia, lethargy, dehydration, general weakness, ‘personality change’, phototoxicity, depression, seizures and hyperkalaemia were also reported (Gehring et al. 1971; Hall et al. 1993; Messinger and Beale 1993; Scott et al. 1993; Kunkle et al. 1995; Tjälve 1997; Rubin et al. 1998; Trapp et al. 2005; Trepanier 2015).

#### PICO 4

Are adverse events more common in cats and dogs treated with TMS for longer duration (> 7 days) in comparison to shorter duration (≤ 7 days)?

No trials comparing AE after short vs. long treatment duration were identified. Among dogs with severe AE or KCS, treatment duration at AE onset was > 7 days in 66 cases and ≤ 7 days in 22 cases.

For severe AE and KCS in dogs, the median (range) treatment duration until onset of the adverse event was 27 days (8–485 days) for longer treatment duration (> 7 days) and 5.5 days (0.5–7 days) for shorter treatment duration (≤ 7 days). Within the shorter-duration group, onset of AE occurred within 7 days in 9 of 22 individuals. In addition, one dog was reported to develop arthropathy after a “few days” of treatment, and two dogs were reported to develop arthropathy within 16 h after re-challenge with TMS.

For mild AE in dogs, treatment duration until onset of the adverse event exceeded 7 days in 25 individuals and was 7 days or less in 10 individuals. The median (range) treatment duration until onset of mild adverse events was 13 days (8–60 days) for longer treatment duration (> 7 days) and 3 days (0.5–7 days) for shorter treatment duration (≤ 7 days). Mild AE were also reported to occur “instantly” in 13 individuals and within “few days” in one individual.

In two studies, one experimental and one clinical study, groups of dogs treated with TMS were followed over time (Seena et al. 2005; Anyogu et al. 2018). No AE were reported during the 4-week follow-up period in the clinical study (Seena et al. 2005). In the experimental study, mild adverse events (dullness and anorexia) were reported after 18 days of TMS treatment (Anyogu et al. 2018).

In cats, the treatment duration reported for any severe AE were 10 and 13 days. For mild AE, salivation and regurgitation were reported in association with drug administration, and hypothyroidism was reported after three days of treatment in one individual.

The treatment duration until onset of AE for dogs and cats is summarized in Supplementary Table 4.

## Discussion

This systematic review identified 110 publications reporting TMS use and/or adverse events (AE) associated with TMS, addressing four prespecified PICO questions. The aims were to identify which AEs are documented to be associated with TMS, how frequently these AEs occur, and whether any patterns related to breed, dose, or treatment duration could be identified.

A pairwise meta-analysis including eight trials did not show a clinically meaningful difference in the number of AEs between TMS and other antimicrobials in dogs. The absolute risk difference showed that dogs treated with TMS had 23 fewer AEs per 1000 treated dogs (95% CI ranging from 45 fewer to 8 more) compared with dogs treated with other antimicrobials, with low certainty evidence.

In cats, the absolute risk difference suggested 35 more AEs per 1000 treated cats (95% CI ranging from 99 fewer to 324 more); however, this estimate was based on a single imprecise study in which the confidence interval crossed three clinical thresholds, resulting in very low certainty evidence.

All reported AEs were mild and did not persist for more than two days. The most commonly reported mild AEs in dogs and cats treated with TMS were related to the gastrointestinal system (vomiting, diarrhoea, inappetence). Four dogs treated with TMS had signs of “eye irritation,” which could have represented early keratoconjunctivitis sicca (KCS), but the signs were not persistent and were therefore interpreted as mild.

The observed risk difference of 23 fewer AEs per 1000 dogs represents a trivial effect according to the a priori defined thresholds, which are intended to indicate effects too small to influence clinical decision-making. Even if the AEs had been severe, the effect would still have been considered trivial, as the predefined threshold for a small effect for severe AEs was 50 AEs per 1000 animals. A limitation of the present thresholds is that they were chosen by the authors alone. The results might have differed if a broader group of stakeholders had been involved; however, the chosen approach prioritizes transparency and allows readers to apply their own interpretations of what constitutes a trivial, small, moderate, or large effect size.

The overall certainty of evidence was low for the pairwise comparison in dogs and very low for cats. Low certainty indicates that the true effect may be markedly different from the estimated effect, while very low certainty indicates that the true effect is probably markedly different from the estimate (Guyatt et al. 2025).

The certainty of evidence would likely have been higher if study authors had explicitly reported the absence of AEs. For dogs, this was done in only two of the eight publications included in the pairwise meta-analysis (Kunkle et al. 1995; Clare et al. 2014). The primary aim of Kunkle et al. (1995) was to investigate owner-reported AEs, which likely explains the higher number of reported events, whereas treatment efficacy was the primary outcome in the remaining studies. Clare et al. (2014) explicitly reported that no AEs occurred, whereas the other studies did not report AE outcomes at all, increasing the risk of bias due to suspected underreporting.

A similar pattern was observed in the proportional meta-analysis in PICO 2, where only 25% (8/31) of studies reported AEs, contributing to the very low certainty of evidence for this analysis as well.

The estimated incidence of mild AEs in dogs treated with TMS was approximately 2%, and the estimated incidence of severe AEs was 0.2%. As this is a systematic review including all identified studies, the results cannot be directly compared with other individual studies or clinical trials.

However, the authors have also analysed retrospective data from 100 dogs treated with TMS (unpublished data), of which only two dogs (2%) developed mild AEs, corresponding closely to the present estimates. The risk of AEs is also similar in humans, where mild AEs are reported to occur in approximately 1% of treated individuals and severe AEs in 0.1% (Anonymous 2021; Anonymous 2023). This suggests that the estimated incidence of approximately 2% may not be far from reality despite the very low certainty of evidence; nevertheless, well-designed prospective trials with standardized AE reporting are required to establish this more robustly.

The 110 publications included in this review were published between 1943 and 2024, covering almost 80 years of TMS use in dogs and cats. Neither prescription data nor total numbers of treated animals during this period were available for inclusion. However, it is reasonable to assume that a large number of dogs and cats have been treated with TMS during this time.

Currently, TMS account for approximately 4.5% of veterinary antibiotic prescriptions, whereas enrofloxacin—considered critically important for human medicine—accounts for approximately 14% of treatments (Anonymous 2020; Anonymous 2024b; Anonymous 2024a). In this context, the number of reported severe AEs is likely to be relatively low, which is supported by the estimated incidence of severe events of approximately 0.2%. Sulfadiazine and sulfamethoxazole were the sulfonamides most frequently implicated in reported AEs, which is unsurprising given that these are likely the most commonly prescribed sulfonamides for dogs and cats. In the absence of prescription data linked to reported AEs, this explanation remains plausible but cannot be confirmed.

A total of 80 observational studies described AEs in further detail. Twenty-two studies described KCS in 107 dogs, and severe AEs were documented in 64 studies including 245 dogs and 2 cats. Mild AEs were reported in 23 studies involving 92 dogs and 15 cats, although dogs and cats were sometimes reported in combined groups without further specification. This should not be interpreted as severe AEs being more common than mild AEs in clinical practice; rather, severe AEs are more likely to be reported and published than mild events such as transient gastrointestinal signs or urticaria.

Scientific publication is not equivalent to pharmacovigilance. Between July 2024 and July 2025, 90 reports of AEs in dogs and cats following sulfamethoxazole treatment were registered in EudraVigilance, the European database of suspected adverse drug reaction reports (Anonymous 2025). Underreporting of AEs in veterinary medicine has also been documented; in a population of veterinarians, only 20% reported all suspected AEs and 20% reported none (Mount et al. 2021).

Most studies contributing to PICO 3 and 4 were retrospective case reports or case series, precluding causal inference regarding treatment duration, dosage, or breed predisposition.

Within these constraints, severe AEs were reported approximately three times more often after long treatment duration (≥ 7 days) than after short duration (< 7 days). This pattern is biologically plausible, as immune-mediated AEs typically require a delay of at least five days before onset of clinical signs (Trepenier et al. 2003; Noli et al. 1995). Such events are therefore unlikely to be captured within a treatment duration of seven days, which represents both a limitation of the chosen threshold for short treatment duration and an argument to limit TMS treatment duration when clinically feasible. The pattern between longer treatment duration and severe AEs was most pronounced for KCS, which was also the most frequently reported severe AE. KCS was reported more frequently in smaller breeds, consistent with previous observations (Campbell 1999). This finding should be interpreted cautiously due to the high proportion of mixed-breed dogs with unknown body size. One possible explanation is that precise dosing may be more difficult to achieve in smaller dogs when using tablets of fixed strength administered per os, potentially resulting in higher doses per kilogram body weight and an increased incidence of KCS. However, in this review it was not possible to determine whether higher doses were overrepresented among dogs with KCS due to incomplete reporting of individual doses.

Another observed pattern was an overrepresentation of Doberman Pinschers developing polyarthritis (10/45 cases). Variability in the cytochrome b5 reductase gene has been associated with sulfonamide hypersensitivity, and Doberman Pinschers are overrepresented for this genetic variant (Funk-Keenan et al. 2012; Reinhart et al. 2018). Samoyeds and Miniature Schnauzers have previously been reported to be overrepresented among dogs with TMS-associated AEs (Trepanier et al. 2003), but this could not be confirmed in the present review.

A key limitation of the included studies was the lack of consistent diagnostic criteria for AEs. The diagnosis of KCS is based on consistent clinical signs and reduced aqueous tear production measured by the Schirmer tear test (Haeussler 2019). In this review, reports of KCS included both peer-reviewed case reports and letters to the editor describing observations without full peer review (e.g., Aguirre 1973), and decreased tear flow alone was accepted as diagnostic KCS (Marino and Jaggy 1993).

Only irreversible KCS was classified as a severe AE. In some dogs, improved but not normalized tear function was reported; these dogs were not considered recovered due to insufficient information, although recovery after the observation period cannot be excluded. Elevated liver enzyme activities following TMS therapy were accepted as markers of hepatic AEs (Noli et al. 1995). These assumptions may have resulted in an overestimation of the frequency of severe AEs.

## Conclusion

Severe AEs, including immune-mediated conditions, hepatic necrosis, and irreversible KCS, have been reported in dogs treated with TMS, but the estimated incidence was low (approximately 0.2%) and based on very low certainty evidence. Mild AEs occur more frequently (approximately 2.3%) in dogs; however, compared with other antimicrobials, TMS treatment was associated with 23 fewer AEs per 1000 treated dogs (95% CI from 45 fewer to 8 more), while a higher incidence was observed in cats based on a single study. The certainty of evidence was low for dogs and very low for cats, largely due to suspected underreporting. Based on the available evidence, treatment with TMS appears to be associated with few serious adverse events in dogs and cats.

## Supplementary Information

Below is the link to the electronic supplementary material.

Supplementary Material 1 (DOCX 59.6 KB)

Supplementary Material 2 (DOCX 32.0 KB)

Supplementary Material 3 (DOCX 274 KB)

## Data Availability

No datasets were generated or analysed during the current study.
