# Supplementary material for: Adverse events of trimetroprim-sulphonamide treatment of cats and dogs: a systematic review
Source: Vet Res Commun. 2026 Mar 25;50(3):224. doi: 10.1007/s11259-026-11143-1 (PMC13018003; doi:10.1007/s11259-026-11143-1)
Supplement: Supplementary file 1 — Supplementary Material 1 (DOCX 59.6 KB) [file 11259_2026_11143_MOESM1_ESM.docx]

Supplementary table 1. A summary of findings table for PICO 1 for adverse events in dogs and cats.

| **TMS compared to Other antimicrobials for treating infections in dogs**  **Bibliography:** | | | | | | | | | | | |
| --- | --- | --- | --- | --- | --- | --- | --- | --- | --- | --- | --- |
| **Certainty assessment** | | | | | | | **Summary of findings** | | | | |
| **Participants (studies) Follow-up** | **Risk of bias** | **Inconsistency** | **Indirectness** | **Imprecision** | **Publication bias** | **Overall certainty of evidence** | **Study event rates (%)** | | **Relative effect (95% CI)** | **Anticipated absolute effects** | |
|  |  |  |  |  |  |  | **With Other antimicrobials** | **With TMS** |  | **Risk with Other antimicrobials** | **Risk difference with TMS** |
| **Adverse events (dogs)** | | | | | | | | | | | |
| 1541 (8 non-randomised studies) | very serious^a^ | not serious | not serious | not serious | none | ⨁◯◯◯ Very low^a^ | 85/875 (9.7%) | 36/666 (5.4%) | **RR 0.76** (0.54 to 1.08) | 85/875 (9.7%) | **23 fewer per 1,000** (from 45 fewer to 8 more) |
| **Adverse events (cats)** | | | | | | | | | | | |
| 149 (1 non-randomised study) | very serious | not serious | not serious | extremely serious | none | ⨁◯◯◯ Very low | 27/125 (21.6%) | 6/24 (25.0%) | **RR 1.16** (0.54 to 2.50) | 27/125 (21.6%) | **35 more per 1,000** (from 99 fewer to 324 more) |

**CI:** confidence interval; **RR:** risk ratio

Supplementary table 2. Number of adverse events, proportions, weight and confidence intervals and random and fixed effect for the proportional meta-analysis for PICO 2.

| Study | Sample size | Proportion (%) | 95% CI | Weight (%) | |
| --- | --- | --- | --- | --- | --- |
|  |  |  |  | Fixed | Random |
| Altreuther *et al.* 2011 | 26 | 0.0 | 0.0 to 13.2 | 0.7 | 2.9 |
| Barber and Trees 1996 | 10 | 30.0 | 6.7 to 65.2 | 0.3 | 1.8 |
| Berger *et al.* 1995 | 33 | 15.1 | 5.1 to 31.9 | 0.9 | 3.2 |
| Bourdeau 1998 | 61 | 0.0 | 0.0 to 5.9 | 1.6 | 3.9 |
| Brahmstaedt *et al.* 1983a | 111 | 1.8 | 0.2 to 6.4 | 2.9 | 4.4 |
| Brahmstaedt *et al.* 1983b | 355 | 0.0 | 0.0 to 1.0 | 9.3 | 4.9 |
| Brunnthaler 1977 | 45 | 0.0 | 0.0 to 7.9 | 1.2 | 3.5 |
| Cannon 1976 | 23 | 0.0 | 0.0 to 14.8 | 0.6 | 2.7 |
| Chretin *et al.* 2007 | 36 | 0.0 | 0.0 to 9.7 | 1.0 | 3.3 |
| Clare *et al.* 2014 | 20 | 0.0 | 0.0 to 16.8 | 0.6 | 2.6 |
| Durr 1976 | 100 | 0.0 | 0.0 to 3.6 | 2.6 | 4.3 |
| Eads 1949 | 54 | 0.0 | 0.0 to 6.6 | 1.4 | 3.7 |
| England *et al.* 2007 | 22 | 0.0 | 0.0 to 15.4 | 0.6 | 2.7 |
| Fernando 1956 | 14 | 0.0 | 0.0 to 23.2 | 0.4 | 2.1 |
| Gehring *et al.* 1971 | 152 | 0.7 | 0.02 to 3.6 | 4.0 | 4.6 |
| Hall *et al.* 1993 | 21 | 0.0 | 0.0 to 16.1 | 0.6 | 2.6 |
| Hardie and Barsanti 1982 | 12 | 0.0 | 0.0 to 26.5 | 0.3 | 2.0 |
| Itoh and Muroaka 2002 | 34 | 0.0 | 0.0 to 10.3 | 0.9 | 3.2 |
| Kose *et al.* 2021 | 22 | 0.0 | 0.0 to 15.4 | 0.6 | 2.7 |
| Kunkle *et al.* 1995 | 196 | 18.4 | 13.2 to 24.5 | 5.1 | 4.7 |
| Lefkaditis 2005 | 30 | 0.0 | 0.0 to 11.6 | 0.8 | 3.1 |
| Ling *et al.* 1984 | 84 | 0.0 | 0.0 to 4.3 | 2.2 | 4.1 |
| Messinger and Beale 1993 | 45 | 6.7 | 1.4 to 18.3 | 1.2 | 3.5 |
| Noli *et al.* 1995 | 2000 | 0.3 | 0.08 to 0.6 | 52.3 | 5.1 |
| Patra and Tripathy 1986 | 32 | 0.0 | 0.0 to 10.9 | 0.9 | 3.2 |
| Samuel *et al.* 1990 | 14 | 0.0 | 0.0 to 23.2 | 0.4 | 2.1 |
| Scott *et al.* 1993 | 20 | 5.0 | 0.1 to 24.9 | 0.6 | 2.6 |
| Seemanthini and Vinodkumar 2016 | 12 | 0.0 | 0.0 to 26.5 | 0.3 | 2.0 |
| Thrusfield *et al.* 1991 | 190 | 0.0 | 0.0 to 1.9 | 5.0 | 4.7 |
| Tripathy 1990 | 10 | 0.0 | 0.0 to 30.9 | 0.3 | 1.8 |
| Younas *et al.* 2014 | 15 | 0.0 | 0.0 to 21.8 | 0.4 | 2.2 |
| Total (fixed effects) | 3799 | 0.8 | 0.5 to 1.1 | 100.0 | 100.0 |
| Total (random effects) | 3799 | 1.8 | 0.7 to 3.3 | 100.0 | 100.0 |

Supplementary table 3. Breeds and substances reported in conjunction with potentiated sulphonamide adverse events in dogs and cats. n-i = number of individuals, n-s = number of studies substance combinations were reported in, NR = not reported

| Keratoconjunctevitis sicca | | | | | | |
| --- | --- | --- | --- | --- | --- | --- |
| Breed | n-i | Substances | n-i | n-s | | |
| Cocker spaniel | 9 | Sulfadiazine-trimethoprim | 39 | 7 | | |
| Geman Shepherd | 9 | Sulfasalazine | 29 | 3 | | |
| Dachshound | 6 | Sulfamethoxazole-trimethoprim | 22 | 5 | | |
| Cavalier King Charles Spaniel | 4 | Sulfisoxazole with phenazopyridine HCL and salicylazosulfapyridine | 1 | 1 | | |
| Miniature Poodle | 4 | Salicylazosulphapyridine | 4 | 4 | | |
| Collie | 4 | Sulfadimethylpyrimidine-trimethoprim | 1 | 1 | | |
| Lhasa Apso | 2 | NR | 23 | 5 | | |
| Airdale Terrier | 2 |  |  |  | | |
| Shetland sheepdog | 2 |  |  |  | | |
| Cairn Terrier | 2 |  |  |  | | |
| Standard Poodle | 2 |  |  |  | | |
| Labrador Retriever | 2 |  |  |  | | |
| Irish Setter | 2 |  |  |  | | |
| Golde Retriever | 1 |  |  |  | | |
| German Pointer | 1 |  |  |  | | |
| Basset | 1 |  |  |  | | |
| Yorkshire Terrier | 1 |  |  |  | | |
| Boxer | 1 |  |  |  | | |
| Elkhound | 1 |  |  |  | | |
| Australian Terrier | 1 |  |  |  | | |
| Siberian husky | 1 |  |  |  | | |
| St Bernard | 1 |  |  |  | | |
| Poodle (size not reported) | 1 |  |  |  | | |
| Brittany | 1 |  |  |  | | |
| English Setter | 1 |  |  |  | | |
| English Springar Spaniel | 1 |  |  |  | | |
| Old English Sheepdog | 1 |  |  |  | | |
| Malteser | 1 |  |  |  | | |
| Corgi | 1 |  |  |  | | |
| Pug | 1 |  |  |  | | |
| Median Poodle | 1 |  |  |  | | |
| Pincher | 1 |  |  |  | | |
| Mixed breeds | 17 |  |  |  | | |
| NR | 20 |  |  |  | | |
| Hepatic necrosis | | | | | | |
| Irish setter | 3 | Sulfamethoxazole-trimethoprim | 10 | | 6 |  |
| Samoyed | 2 | Sulfadiazine-trimethoprim | 9 | | 7 |  |
| Schnauzer | 2 | Sulfatroxazole-trimethoprim | 1 | | 1 |  |
| Chihuahua | 2 | Sulfasalzine | 1 | | 1 |  |
| Golden retriever | 1 | NR | 13 | | 3 |  |
| Hungarian shepard | 1 |  |  | |  |  |
| Maltese terrier | 1 |  |  | |  |  |
| West highland white terrier | 1 |  |  | |  |  |
| Bishon | 1 |  |  | |  |  |
| Vizla | 1 |  |  | |  |  |
| St Bernard | 1 |  |  | |  |  |
| NR | 13 |  |  | |  |  |
| Hematological adverse events | | | | | | |
| Nigerian local breed | 15 | Sulfamethoxazole-trimethoprim | 20 | | 6 |  |
| Miniature poodle | 12 | Sulfadiazine-trimethoprim | 6 | | 5 |  |
| Mongrel | 3 | Sulfatroxazole-trimethoprim | 1 | | 1 |  |
| Golden retreiver | 2 | Sulfaquinoxaline | 25 | | 2 |  |
| Greyhound | 2 | Sulfasalazine | 1 | | 1 |  |
| Irish setter | 1 | NR | 37 | | 2 |  |
| "Bobtail" | 1 |  |  | |  |  |
| Austrailian terrier | 1 |  |  | |  |  |
| Bluetick cornhound | 1 |  |  | |  |  |
| Rotweiler | 1 |  |  | |  |  |
| Basset | 1 |  |  | |  |  |
| Mixed breed | 1 |  |  | |  |  |
| Greyhound | 2 |  |  | |  |  |
| Scottish terrier | 1 |  |  | |  |  |
| Great pyrenees | 1 |  |  | |  |  |
| NR | 57 |  |  | |  |  |
| Polyarthritis/arthropathy | | | | | | |
| Doberman Pincher | 10 | Sulfadiazine-trimethoprim | 23 | | 7 |  |
| Golden Retriever | 4 | Sulfamethoxazole-trimethoprim | 3 | | 2 |  |
| Gorden Setter | 4 | Sulfadimethoxazole-trimethoprim | 1 | |  |  |
| Weimaraner | 3 | Sulfatroxazole-trimetoprim | 1 | | 1 |  |
| Irish Setter | 2 | NR | 27 | | 4 |  |
| Labrador retriever | 2 |  |  | |  |  |
| Enlish Springer Spaniel | 2 |  |  | |  |  |
| German Pointer | 2 |  |  | |  |  |
| Flatcoated Retriver | 1 |  |  | |  |  |
| Cockerspaniel | 1 |  |  | |  |  |
| Pekines | 1 |  |  | |  |  |
| Briard | 1 |  |  | |  |  |
| Greyhound | 1 |  |  | |  |  |
| Giant Schnauzer | 1 |  |  | |  |  |
| Drentse Patriijs Hound | 1 |  |  | |  |  |
| Airdale Terrier | 1 |  |  | |  |  |
| Mixed breed | 1 |  |  | |  |  |
| NR | 7 |  |  | |  |  |
| Other immune mediated adverse events^1^ | | | | | | |
| Doberman | 3 | Sulfamethoxazole-trimethoprim | 9 | | 6 |  |
| Minature Schnauzer | 2 | Sulfadiazine-trimethoprim | 11 | | 5 |  |
| Golden retriver | 2 | Sulfadimetoxine-ormetoprim | 1 | | 1 |  |
| German shephard | 2 | NR | 9 | | 5 |  |
| Great dane | 2 |  |  | |  |  |
| Standard poodle | 2 |  |  | |  |  |
| Fench bulldog | 1 |  |  | |  |  |
| Bouvier des flandres | 1 |  |  | |  |  |
| Hungarian shepard | 1 |  |  | |  |  |
| Maltese terrier | 1 |  |  | |  |  |
| New foundland | 1 |  |  | |  |  |
| Labrador retriever | 1 |  |  | |  |  |
| West highlnad white terrier | 1 |  |  | |  |  |
| Enlish sringer spaniel | 1 |  |  | |  |  |
| Old english sheepdog | 1 |  |  | |  |  |
| Mongrel | 1 |  |  | |  |  |
| Dalmatian | 1 |  |  | |  |  |
| Pembroke welsh corgi | 1 |  |  | |  |  |
| Brittany spaniel | 1 |  |  | |  |  |
| Jack russel | 1 |  |  | |  |  |
| Pincher | 1 |  |  | |  |  |
| mixed breed | 3 |  |  | |  |  |
| NR | 11 |  |  | |  |  |
|  |  |  |  | |  |  |

**^1^**Those adverse events included iatrogen hypothyroid crises, Erythema multiforme, Skin rash, Facial swelling, eye irritation, Skin eruptions and multifocal ulcerations, Pustular and exfoliative dermatitis, (mucocutaneous sloughing and ulceration, Toxic epidermal necrolysis, Vasculitis, Generalized maculopapular dermatitis and Goitrous hypothyroidism

Supplementary table 4. Type of potentiated sulphonamide, dose and therapy duration until onset of adverse events.

| Study | n | Sulfonamide(s) (n) | Dose(s) | TMS therapy duration to AE onset (n) |
| --- | --- | --- | --- | --- |
| KCS | | | | |
| Aguirre 1973 | > 1 | Sulfasoxazole and Salicylazosulfapyridine | NR | 18 h - months |
| Bedford 1985 | 6 | Sulfadiazin | NR | 35 days (1) NR (5) |
| Berger *et al.* 1995 | 5 | Sulfadiazin | 8.6-104 mg/kg SID | 7 days (1), 15 days (1), 21 days (2) and 7 months (1) |
| Collin *et al.* 1986 | 11 | Sulfamethoxazole | 11.7-65.7 mg/kg BID | NR |
| Diehl and Roberts 1991 | 16 | Sulfamethoxazole (6), Sulfadiazin (4), Sulfasalazin (3) and TMS (3) | 11.7-65.7 mg/kg BID | 9-365 days (median=30 days) |
| Frank *et al.* 2005 | 2 | sulfamethoxazole | 14.1-16 mg/kg BID | 21 days (2) |
| Lavergne *et al.* 2008 | NR | TMS | NR | NR |
| Lewis *et al.* 2023 | 1 | sulfamethoxazole | 30 mg/kg | 20 days |
| Marino and Jaggy 1993 | 1 | sulfamethoxazole | 40 mg/kg BID | 15 days |
| Morgan and Bachrach 1982 | 14 | sulfasalazine (13),  TMS (1) | 12.5-65 mg/kg TID (13), 100 mg/kg TID (1) | NR |
| Nuttall and Malham 2004 | 1 | TMS | NR | 3 days |
| Sansom *et al.* 1985 | 13 | Sulfasalazine | 125-500 mg BID | 2 months (2),  5 months (1),  6 months (2),  7 moths (1),  8 months (2), 11months (1), 12 months (3), 16 months (1) |
| Sansom and Barnett 1985 | 3 | salicylsulfapyridin | NR | 5 months (1), 12 months (1), NR (1) |
| Slatter and Blogg 1978 | 14 | sulfadiazine (13), salicylsulfapyridin (1) | NR | 3 days (2),  3 months (3),  4 months (2),  5 months (2),  6 months (3), 7.5 months (1), 12 months (1 |
| Sutton and Roach 1988 | 1 | Sulfadiazine | 12.5 mg/kg BID | 12 days |
| Tarlow *et al.* 2005 | 1 | sulfamethoxazole | 15 mg/kg BID | 21 days |
| Tjalve 1997 | 2 | NR | NR | NR |
| Tjälve 1997 | 8 | sulfadiazine or sulfamethoxazole | NR | NR |
| Tjalve *et al.* 2019 | 1 | sulfamethoxazole | NR | NR |
| Todenhofer 1969 | 8 | Sulfadiazine | NR | NR |
| Trapp *et al.* 2005 | 1 | Sulfadiazine | NR | NR |
| Trepanier *et al.* 2003 | 10 | NR | 23.4-81.4 mg/kg per day | 5-36 days (mean 12.1) |
| Tuntivanich *et al.* 1997 | 1 | sulfadimethylpyrimidine | NR | 2 days |
| Twedt *et al.* 1997 | 1 | sulfamethoxazole | 32 mg/kg BID | 26 days |
| Hepatopathy and hepatic necrosis | | | | |
| Anderson *et al.* 1984 | 1 | Sulfadiazine | NR | 11 days |
| Anonymous 1995 | 1 | Sulfadiazine | NR | 5 days |
| Diehl and Roberts 1991 | 1 | sulfamethoxazole | 29.7 mg/kg BID | ~ 30 days |
| Dodds 1997 | 1 | NR | NR | NR |
| Funk-Keenan *et al.* 2012 | 9 | sulfamethoxazole or sulfadiazine or sulfadidimethoxin | 25.4-68.4 mg/kg per day | 5-36 days |
| Johnson *et al.* 2023 | 2 | sulfamethoxazole | 27 mg/kg (1) 30 mg/kg (1) | 11 days  28 days |
| Lavergne *et al.* 2008 | NR | TMS | NR | NR |
| Messinger and Beale 1993 | 1 | Sulfadiazine | 30-40.7 mg/kg BID | < 12 h |
| Noli *et al.* 1995 | 2  1  1 | sulfadiazine or  sulfatroxazole or  sulfamethoxazole | NR | 9 days (1), 15-30 days (1) 10 days (1), 9 days (1) |
| Rowland *et al.* 1992 | 1 | Sulfadiazine | 30 mg/kg BID | 7-14 days |
| Thomson 1990 | 1 | sulfamethoxazole | 20 mg/kg BID | < 2 days |
| Thornburg 1988 | 2 | Sulfadizine | NR | 1 day |
| Tjalve 1997 | 1 | NR | NR | NR |
| Tjalve *et al.* 2019 | 2 | sulfamethoxazole | NR | 7 days,  < 21 days |
| Trepanier *et al.* 2003 | 11 | NR | 23.4-81.4 mg/kg per day | 5-36 days (mean 12.1) |
| Twedt *et al.* 1997 | 4 | sulfamethoxazol (3) or  sulfadiazine (1) | 18 mg/kg BID, 21 mg/kg BID, 32 mg/kg BID,  53 mg/kg BID | 4 days (1), 7 days (1), 12 days (1), 30 days (1) |
| Pena-Ramos *et al.* 2021 | 1 | Sulfasalzine | 15 mg/kg BID | 1 year |
| idiosyncratic sulfonamide toxicity and iatrogenic hypothyroid crisis | | | | |
| Brenner *et al.* 2009 | 1 | sulfamethoxazole | 40 mg/kg BID | 10 days |
| Trepanier *et al.* 2003 | 1 | sulfamethoxazole | 15 mg/kg BID | 12 days |
| Hematological adverse events | | | | |
| Anyogu *et al.* 2018 | 15 | sulfamethoxazole | 30 mg/kg BID (5) 60 mg/kg BID (5) or  120 mg/kg BID (5) | 7 days |
| Fox *et al.* 1993 | 2 | Sulfadiazine | 25 mg/kg BID and 30-60 mg/kg BID | 10 days (1), 30 days (1) |
| Jeong *et al.* 2023 | 1 | sulfamethoxazole | 15 mg/kg BID | NR |
| McEwan 1992 | 1 | sulfamethoxazole | 28 mg/kg BID | 42 days |
| Noli *et al.* 1995 | 2 | Sulfadiazine (1), sulfatroxazole (1) | NR | 7 days (1), 11 days (1) |
| Osweiler and Green 1978 | 13 | Sulfaquinoxaline | NR | 2-5 days |
| Patterson and Grenn 1975 | 12 | sulfaqunoxaline | NR | 1 day |
| Pena-Ramos *et al.* 2021 | 1 | sulfasalazine | 15 mg/kg BID | 1 year |
| Sullivan *et al.* 1992 | 1 | sulfadiazine | 18.8 mg/kg BID | < 7 days |
| Tarlow *et al.* 2005 | 1 | sulfamethoxazole | 15 mg/kg BID | 21 days |
| Tham *et al.* 2016 | 1 | sulfamethoxazole | 15 mg/kg SID for 20 days, then 23 mg/kg BID | 6 days |
| Trepanier *et al.* 2003 | 20 | NR | 23.4-81.4 mg/kg per day | 5-36 days (mean 12.1) |
| Trimborn and Vick 1992 | 1 | sulfamethoxazole | 30 mg/kg per day | 21 days |
| Weiss and Adams 1987 | 1 | sulfadiazine | 15 mg/kg BID | 16 days |
| Weiss and Klausner 1990 | 1 | sulfadiazine | 14 mg/kg BID | 19 days |
| Artropathy | | | | |
| Funk-Keenan *et al.* 2012 | 2 | sulfamethoxazole or sulfadiazine or sulfadidimethoxin | 25.4-68.4 mg/kg per day | 5-36 days |
| Gray 1990 | 13 | TMS | NR | NR |
| Giger *et al.* 1985 | 6 | sulfadiazine | 27.4 mg/kg BID (1), NR (5) | 10-21 days (5) 19 days (1) |
| Grondalen 1987 | 7 | TMS | NR | 8-20 days |
| Harvey 1987 | 2 | sulfadiazine | 30 mg/kg BID | 8 days |
| Lavergne *et al.* 2008 | NR | TMS | NR | NR |
| Lees *et al.* 1986 | 1 | sulfadiazine | 36 mg/kg BID | 6 days |
| Little and Carmichael 1990 | 1 | sulfadiazine | NR | 7 days |
| Medleau *et al.* 1990 | 1 | sulfadiazine | 12 mg/kg BID | < 3 days |
| Noli *et al.* 1995 | 7 | sulfadiazine (6), sulfatroxazole (1) | NR | 7 days (2), 9 days (1), 11 days (1), 12 days (1), 14 days (1), NR (1) |
| Taksdal 1987 | 4 | sulfadiazine | 480 mg TID (1), NR (3) | ~ 10 days (1), ~ 20 days (1), NR (2). |
| Trepanier *et al.* 2003 | 6 | NR | 23.4-81.4 mg/kg per day | 5-36 days (mean 12.1) |
| Werner and Bright 1983 | 2 | sulfadiazine | NR | 1-16 hours* |
| Whur 1987 | 1 | sulfamethoxazole | 12 mg/kg TID | “few days” |
| Dermatologic adverse events | | | | |
| Delmage and Payne-Johnson 1991 | 1 | sulfamethoxazole | 10 mg/kg BID | 3 days |
| Kunkle *et al.* 1995 | 1 | sulfadiazine | NR | NR |
| Lavergne *et al.* 2008 | NR | suladimethoxine or  sulfametoxazole or  sulfadiazine | NR | ≥ 5 days |
| Marino and Jaggy 1993 | 1 | TMS | 40 mg/kg BID | NR |
| Medleau *et al.* 1990 | 6 | sulfamethoxazole (4) sulfadiazine (2) | 12 mg/kg (1), 15 mg/kg BID(1), 18 mg/kg BID (1), 22 mg/kg BID (1), 24 mg/kg BID (1), 32 mg/kg SID (1) | 3 days (1), 7 days (1), 8 days (1), 10 days (1),12 days (1), < 30 days (1) |
| Noli *et al.* 1995 | 13 | Sulfadiazine (9), sulfatroxazole (2), succinylsulfathiazole (1), sulfamethoxazole (1) | NR | 9 days (1),10 days (2), 11 days (1), 12 days (2), 13 days (3), 14 days (1), 28 days (1), 15-30 days (1), NR (1) |
| Nuttall and Malham 2004 | 1 | TMS | NR | 3 days |
| Scott and Miller 1999 | 8 | sulfadiazine (6), sulfamethoxazole (1), sulfadimethozine (1) | NR | 7-21 days |
| Scott *et al.* 1986 | 1 | sulfamethoxazole | NR | 2 months |
| Tjalve 1997 | 1 | NR | NR | NR |
| Tjalve *et al.* 2018 | 1 | sulfamethoxazole | NR | “few days” |
| Tjalve *et al.* 2017 | 1 | sulfamethoxazole | NR | NR |
| Tjälve 1997 | 1 | NR | NR | NR |
| Trapp *et al.* 2005 | 1 | sulfadiazine | NR | NR |
| Trepanier *et al.* 2003 | 4 | NR | 23.4-81.4 mg/kg per day | 5-36 days (mean 12.1) |
| Werner and Bright 1983 | 1 | sulfadiazine | NR | 1-16 h |
| Other severe adverse events | | | | |
|  |  |  |  |  |
| Funk-Keenan *et al.* 2012 | 1 | sulfamethoxazole or sulfadiazine or sulfadidimethoxin | 25.4-68.4 mg/kg per day | 5-36 days |
| Kunkle *et al.* 1995 | 41 | sulfadiazine | NR | NR |
| Lavergne *et al.* 2008 | NR | suladimethoxine or  sulfametoxazole or  sulfadiazine | NR | ≥ 5 days |
| Noli *et al.* 1995 | 6 | Sulfadiazine (5), sulfatroxazole (1), | NR | 7 days (2), 13 days (1), 14 days (1), NR (2) |
| Nuttall and Malham 2004 | 1 | TMS | NR | 3 days |
| Trapp *et al.* 2005 | 1 | sulfadiazine | NR | NR |
| Trepanier *et al.* 2003 | 3 | NR | 23.4-81.4 mg/kg per day | 5-36 days (mean 12.1) |
| Other mild adverse events | | | | |
| Anderson *et al.* 1984 | 1 | sulfadiazine | NR | 11 days |
| Anonymous 1995 | 3 | Sulfadiazine | NR | NR |
| Barber and Trees 1996 | 2 | sulfamethoxazole | 15 mg/kg (1), 20 mg/kg (1) | NR |
| Brahmstaedt *et al.* 1983a | 13 | sulfamerazine | 24 mg/kg BID | Instantly |
| Cannon 1976 | NR (“some cats” | sulfamerazine | ~ 30 mg/kg SID | NR |
| Frank *et al.* 1989 | 1 | sulfadiazine | 16 mg/kg BID | 13 days |
| Frank *et al.* 2005 | NR | sulfamethoxazole | 14-16 mg/kg BID | ≥ 21 days |
| Gehring *et al.* 1971 | 1 | sulfadoxine | 15 mg/kg | 1 day |
| Gookin *et al.* 1999 | 1 | sulfadiazine | 24 mg/kg BID | 40 days |
| Hall *et al.* 1993 | NR | sulfamethoxazole | 30 mg/kg BID | NR |
| Halman *et al.* 2024 | 1 | sulfadoxin and sulfadiazine | 25-30 mg/kg BID and 32 mg/kg BID | 3 days |
| Kunkle *et al.* 1995 | 42 (36 dogs, 6 cats) | sulfadiazine | NR | NR |
| Lavergne *et al.* 2008 | NR | TMS | NR | NR |
| Marino and Jaggy 1993 | 1 | TMS | 40 mg/kg BID | 15 days |
| Messinger and Beale 1993 | 2 | sulfadiazine (1), sulfadimethoxine (1) | 30.2-40.7 mg/kg BID (1), 55-80 mg/kg SID (1) | NR |
| Nuttall and Malham 2004 | 1 | TMS | NR | 3 days |
| Rubin *et al.* 1998 | 1 | sulfamethoxazole | 33 mg/kg | 30 days |
| Scott *et al.* 1993 | 1 | sulfadimethoxine | 55 mg/kg SID, then 27.5 mg/kg SID | 2 days |
| Seelig *et al.* 2008 | 1 | sulfamethoxazole | 30 mg/kg BID | 35 days |
| Taeymans and O'Marra 2009 | 1 | sulfamethoxazole | NR | NR |
| Tjälve 1997 | NR | sulfadiazine, sulfamethoxazole | NR | NR |
| Tjalve *et al.* 2019 | 1 | sulfadiazine | NR | 11 days |
| Torres *et al.* 1996 | 1 | Sulfadiazone | 25 mg/kg BID | NR |
| Trepanier *et al.* 2003 | 2 | NR | 23.4-81.4 mg/kg per day | 5-36 days (mean 12.1) |
| Vasilopulos *et al.* 2005 | 1 | sulfadimethoxine | 60 mg/kg SID, then 30 mg/kg SID | 26 days |
| Wilkinson 1977 | NR (a number of cats) | sulfadimethoxine | 50 mg/kg SID | NR |
| Williamson *et al.* 2002 | 6 | sulfamethoxazole | 26.5-31.3 mg/kg BID | 7-21 days |
