# Supplementary material for: Adverse events of trimetroprim-sulphonamide treatment of cats and dogs: a systematic review
Source: Vet Res Commun. 2026 Mar 25;50(3):224. doi: 10.1007/s11259-026-11143-1 (PMC13018003; doi:10.1007/s11259-026-11143-1)
Supplement: Supplementary file 2 — Supplementary Material 2 (DOCX 32.0 KB) [file 11259_2026_11143_MOESM2_ESM.docx]

# ADVERSE EFFECTS OF TRIMETROPRIM-SULPHONAMIDE TREATMENT OF CATS AND DOGS: A SYSTEMATIC REVIEW PROTOCOL

Karolina Scahill^1*^, Lena Pelander^2^, Isabelle Nesterud^3^, Britt-Marie Bergquist^3^, Mia Hedlund^4^, Carl Ekstrand^4^

^1^University of Edinburgh, College of Medicine and Veterinary Medicine, Edinburgh, United Kingdom

^2^ Department of Clinical Sciences, Swedish University of Agricultural Sciences, Uppsala, Sweden

^3^ SLU University Library, Swedish University of Agricultural Sciences, Uppsala, Sweden

^4^ Department of Animal Biosciences, Swedish University of Agricultural Sciences, Uppsala, Sweden

^*^Corresponding author: [karolinascahill@gmail.com](mailto:karolinascahill@gmail.com)

**Protocol and registration**

This protocol was written using the Preferred Reporting Items for Systematic Reviews and Meta-Analysis for Systematic Reviews (PRISMA-P) reporting guidelines (Moher et al., 2015). It will be published on the slu.se website and will be registered at SYREAF ([www.syreaf.org](http://www.syreaf.org)).

**Funding:** The study is partly funded by The Greater Stockholm Veterinary Care Foundation, which is a non-profit foundation.

**INTRODUCTION**

**Rationale**

The World Health Organization (WHO) has advocated that antibiotics such as monobactams, lipopeptides and carbapenams, that are considered critically important to human health should not be used in animals (Anonymous, 2024). Consistent with WHO, the European Medicines Agency has published recommendations for use of antibiotics in veterinary medicine, including recommended first line antibiotics (Anonymous, 2020). Fluoroquinolones are third line antibiotics that only should be used in veterinary medicine when no alternatives are available but is the second most prescribed antimicrobial in companion animals according to the World Organisation for Animal Health (Anonymous, 2024). An example of first line antibiotics with activity against both gram-positive and gram-negative bacteria are potentiated sulphonamides (TMS). Their broad antibacterial spectrum, bactericidal effects and high distribution to body tissues could make them feasible for use in soft tissue infections when gram-negative infections can be suspected, for example pyelonephritis, prostatitis and sepsis in dogs and cats. Adverse effects and hypersensitivity reactions, such as immune-mediated disease, has been described in association with TMS treatment in cats and dogs (Noli et al., 1995; Trepanier et al, 2003). A perceived high risk of severe adverse events likely contributes to TMS being underprescribed by veterinary practitioners that might choose to prescribe fluoroquinolones instead. The aim of this systematic review is to investigate the risk of adverse effects of TMS treatment in cats and dogs.

# Objectives/research questions

# What adverse effects have been reported in TMS treatment of cats and dogs?

# Are adverse effects more common in cats and dogs treated with TMS in comparison to other antimicrobials?

# Are adverse effects more common in cats and dogs treated with TMS for longer duration in comparison to shorter duration?

# What proportion of TMS treatments in dogs and cats result in adverse effects?

# PICOS/PICC

| # | Population | Intervention | Comparator or context | Outcome |
| --- | --- | --- | --- | --- |
| 1 | Cats and dogs | TMS | Descriptive (no comparator) | Adverse effects |
| 2 | Cats and dogs | TMS | Other antimicrobials | Adverse effects |
| 3 | Cats and dogs | TMS long duration | TMS short duration | Adverse effects |
| 4 | Cats and dogs | TMS | Proportion (no comparator) | Adverse effects |

Table 1 shows included population, intervention, comparator and outcome (PICO).

**METHODS**

**Eligibility criteria**

All studies that report adverse events of TMS and/or trials using TMS will be included. There will be no date restrictions. Study design eligibility varies between different PICOs (table 2).

| PICO | Inclusion | Exclusion |
| --- | --- | --- |
| 1 | Randomized controlled trials, all observational studies (controlled and uncontrolled), case reports, conference abstracts, editorial letters that report adverse events for TMS | Reviews (but references screened) |
| 2 | Randomized controlled trials, controlled cohorts using TMS and another antimicrobial | Reviews, case reports, trials without controls, conference abstracts, editorial letters, other reports |
| 3 | Randomized controlled trials, controlled cohorts using TMS | Reviews, case reports, trials without controls, conference abstracts, editorial letters, other reports |
| 4 | Randomized controlled trials, observational studies, case series (>10 animals) using TMS | Reviews, case reports, conference abstracts, editorial letters, other reports |
| 1,2,3,4 | Experimental disease studies (disease conditions such as induced urinary tract infection). | Experimental studies and safety studies with exceptionally high doses that are not used in practice (>50 mg/kg). All experimental studies on other species than cats and dogs |
| 1,2,3,4 | Swedish, English, German, Danish, Norwegian | Other languages |

Table 2 shows inclusion and exclusion criteria for the different PICOs

**Information sources and search strategy**

Searches were conducted between 2024-10-24 and 2024-10-30 in the CABI databases: Cab abstracts, Medline, Web of Science Core Collection, PubMed and Scopus. The total number of hits was 9014 from all databases. Duplicate removal was performed in Endnote, according to the Karolinska Institutet Library's method ([https://kib.ki.se/node/1379. 387](https://kib.ki.se/node/1379.%20387)) duplicates were removed and 5143 hits were ultimately left. Grey literature will not be explored.

| CABI: Cab abstracts, Web of Science/Clarivate  Date of search: 2024-10-24  Number of hits: 1597 | Field codes  TS = title, abstract, descriptors, Cabicodes, identifiers  * = truncation of word for alternate endings |
| --- | --- |

| # | Search string | Number of hits |
| --- | --- | --- |
| 1 | TS=(cat OR cats OR feline* OR dog OR dogs OR canine*) | 387,643 |
| 2 | TS=(trimethoprim OR sulphonamid* OR sulfonamid* OR sulfamethoxazol* OR sulphamethoxazol* OR sulphadiazin* OR sulfadiazin* OR sulfadimethoxin* OR sulphadimethoxin* OR sulfadimedin* OR sulphadimedin* OR sulfadoxin* OR sulphadoxin* OR sulfamethoxypyridazin* OR sulphamethoxypyridazin*) | 35,068 |
| 3 | 1 AND 2 | 1,597 |

| Medline, Web of Science/Clarivate  Date of search: 2024-10-24  Number of hits: 2457 | Field codes   - TS = title, abstract, MeSH headings and qualifiers - * = truncation of word for alternate endings |
| --- | --- |

| # | Search string | Number of hits |
| --- | --- | --- |
| 1 | TS=(cat OR cats OR feline* OR dog OR dogs OR canine*) | 598,471 |
| 2 | TS=(trimethoprim OR sulphonamid* OR sulfonamid* OR sulfamethoxazol* OR sulphamethoxazol* OR sulphadiazin* OR sulfadiazin* OR sulfadimethoxin* OR sulphadimethoxin* OR sulfadimedin* OR sulphadimedin* OR sulfadoxin* OR sulphadoxin* OR sulfamethoxypyridazin* OR sulphamethoxypyridazin*) | 105,240 |
| 3 | 1 AND 2 | 2457 |

| PubMed, NCBI  Date of search: 2024-10-30  Number of hits: 58 | Field codes   - No field codes have been used to utilize the database's own mapping to MeSH, etc. |
| --- | --- |

| # | Search string | Number of hits |
| --- | --- | --- |
| 1 | (cat OR cats OR feline OR dog OR dogs OR canine) | 629,706 |
| 2 | (trimethoprim OR sulphonamid OR sulfonamid OR sulphonamide OR sulfonamide OR sulfamethoxazol OR sulphamethoxazol OR sulfamethoxazole OR sulphamethoxazole OR sulphadiazin OR sulfadiazine OR sulphadiazine OR sulfadiazin) | 173,080 |
| 3 | 1 AND 2 | 4,824 |
| 4 | Filter: Species: Other animal Time limit: 2024-01-01 – 2026-01-01 | 58 |

| Scopus  Date of search: 2024-10-24  Number of hits: 3547 | Field codes   - TITLE-ABS-KEY = title, abstract, and keywords - * = truncation of word for alternate endings |
| --- | --- |

| # | Search string | Number of hits |
| --- | --- | --- |
| 1 | TITLE-ABS-KEY (cat OR cats OR feline* OR dog OR dogs OR canine*) | 749,375 |
| 2 | TITLE-ABS-KEY (trimethoprim OR sulphonamid* OR sulfonamid* OR sulfamethoxazol* OR sulphamethoxazol* OR sulphadiazin* OR sulfadiazin* OR sulfadimethoxin* OR sulphadimethoxin* OR sulfadimedin* OR sulphadimedin* OR sulfadoxin* OR sulphadoxin* OR sulfamethoxypyridazin* OR sulphamethoxypyridazin*) | 166,322 |
| 3 | 1 AND 2 | 3,547 |

| Web of Science Core collection, Web of Science  Date of search: 2024-10-24  Number of hits: 1355 | Field codes   - TS/Topic = title, abstract, author keywords and keywords plus - * = truncation of word for alternate endings |
| --- | --- |

| # | Search string | Number of hits |
| --- | --- | --- |
| 1 | TS=(cat OR cats OR feline* OR dog OR dogs OR canine*) | 664,182 |
| 2 | TS=(trimethoprim OR sulphonamid* OR sulfonamid* OR sulfamethoxazol* OR sulphamethoxazol* OR sulphadiazin* OR sulfadiazin* OR sulfadimethoxin* OR sulphadimethoxin* OR sulfadimedin* OR sulphadimedin* OR sulfadoxin* OR sulphadoxin* OR sulfamethoxypyridazin* OR sulphamethoxypyridazin*) | 74,338 |
| 3 | 1 AND 2 | 1,355 |

**Data management and selection process**

Screening will be done in the systematic review tool covidence (www.covidence.org) and will be performed in duplicate independently by two reviewers, conflicts will be solved through discussion or by a third reviewer.

Title and abstract screening

In the first level, the reviewers will independently evaluate the protocols relevance by using the following screening question:

1. Does the abstract include TMS side effects or TMS treatment of cats and dogs?

Yes= include, No= exclude, Uncertain: include for full text evaluation

1. Is the abstract written in English, Swedish, Norwegian, Danish or German?

Yes= include, No= exclude

Full text screening

In the second level, the reviewers will independently evaluate the protocols relevance by using the following screening question:

1. Does the full text report TMS side effects (any study design and number of animals) or TMS treatment of at least ten cats and dogs?

Yes= include, No= exclude

1. Is the full text written in English, Swedish, Norwegian, Danish or German?

Yes=include, No=exclude (if the abstract includes the relevant information in one of the included languages it can however be included)

1. Does the study include TMS dosages (>50 mg/kg) that are not used in practice?

Yes=exclude, No=include

**Data collection process and items**

Data will be extracted in Excel spreadsheet (<https://office.microsoft.com/excel>) in duplicate independently by two reviewers. Conflicts will be solved through discussion or by a third reviewer. The following data will be extracted: 1) General information: first author, title, journal, year of publication, funding, country of study (the country of the corresponding author will be chosen if the country is not disclosed, PICO 1-4, 2) Study design: RCT, observational, case report, retrospective/prospective, multi or single centre, experimental infection (yes/no), setting (shelter/owned animals/laboratory animals), 3) Treatment information: substance (intervention and comparator), total animals treated, total animals with adverse effects, dose, duration of treatment, time to onset of AE (from start of treatment), 4) Adverse event information: hypersensitivity, KCS, non-hypersensitivity, mortality (due to AE), breed and sex of animals with hypersensitivity reaction and KCS.

**Outcomes and prioritization**

The main outcome are adverse events that will be sub-categorized in severe and mild reactions (table 3).

| **Severe** | **Mild** |
| --- | --- |
| 1. Immune-mediated disease (often called hypersensitivity reactions) such as thrombocytopenia and polyarthritis, mucocutaneous ulceration 2. Anaphylactic chock 3. Hepatic necrosis, acute kidney injury 4. Irreversible KCS and other irreversible conditions 5. Mortality due to adverse effects | 1. Mild dermatological reactions 2. Transient swelling/urticaria 3. Gastrointestinal disease (diarrhoea, vomiting) 4. Lethargy/mild stiffness 5. Polyuria/polydipsia 6. Reversible KCS |

Table 3 shows examples (but is not limited to) of severe and mild adverse reactions

**Data analysis and presentation of results**

PICO 1 will be synthesized descriptively. A pairwise meta-analysis will be performed for PICO 2 and 3 by using the RevMan web tool (<https://revman.cochrane.org>). A proportional meta-analysis will be performed for PICO 4. If pairwise and proportional meta-analysis is not possible descriptive data will be reported instead. Risk of bias will be assessed for PICO 2-4 with the RoB 2 tool (<https://www.riskofbias.info/welcome/rob-2-0-tool/current-version-of-rob-2> ) for randomized studies and ROBINS-I V2 tool (<https://www.riskofbias.info/welcome/robins-i-v2>) will be used for observational studies. GRADEpro (<https://www.gradepro.org/>) will be used to generate summary of findings tables and to calculate absolute effects for PICO 2 and 3. The Grading of Recommendations, Assessment, Development and Evaluation (GRADE) methodology will be used to assess the certainty of evidence for PICO 2-4.

# References

# Anonymous. Categorisation of antibiotics in the European Union. European Medicines Agency. 2020. <https://www.ema.europa.eu/en/documents/report/categorisation-antibiotics-european-union-answer-request-european-commission-updating-scientific-advice-impact-public-health-and-animal-health-use-antibiotics-animals_en.pdf> [Accessed 2025-05-19]

# Anonymous. WHO's List of Medically Important Antimicrobials: a risk management tool for mitigating antimicrobial resistance due to non-human use. Geneva: World Health Organization; 2024. Licence: CC BY-NC-SA 3.0 IGO. <https://cdn.who.int/media/docs/default-source/gcp/who-mia-list-2024-lv.pdf> [Accessed 2025-05-19]

# Anonymous. Annual Report on Antimicrobial Agents Intended for Use in Animals. 8^th^ report. Available from: <https://www.woah.org/app/uploads/2024/05/woah-amu-report-2024-final.pdf> [Accessed 2025-05-19]

# Moher D, Shamseer L, Clarke M, Ghersi D, Liberati A, Petticrew M, Shekelle P, Stewart LA; PRISMA-P Group. Preferred reporting items for systematic review and meta-analysis protocols (PRISMA-P) 2015 statement. Syst Rev. 2015 Jan 1;4(1):1. doi: 10.1186/2046-4053-4-1. PMID: 25554246; PMCID: PMC4320440.

Noli C, Koeman JP, Willemse T. A retrospective evaluation of adverse reactions to trimethoprim-sulphonamide combinations in dogs and cats. Vet Q. 1995 Dec;17(4):123-8. doi: 10.1080/01652176.1995.9694550. PMID: 8751272.

Trepanier LA, Danhof R, Toll J, Watrous D. Clinical findings in 40 dogs with hypersensitivity associated with administration of potentiated sulfonamides. J Vet Intern Med. 2003 Sep-Oct;17(5):647-52. doi: 10.1111/j.1939-1676.2003.tb02495.x. PMID: 14529130.
