# Supplementary material for: Adverse events of trimetroprim-sulphonamide treatment of cats and dogs: a systematic review
Source: Vet Res Commun. 2026 Mar 25;50(3):224. doi: 10.1007/s11259-026-11143-1 (PMC13018003; doi:10.1007/s11259-026-11143-1)
Supplement: Supplementary file 3 — Supplementary Material 3 (DOCX 274 KB) [file 11259_2026_11143_MOESM3_ESM.docx]

| **Section and Topic** | **Item #** | **Checklist item** | **Location where item is reported** |
| --- | --- | --- | --- |
| **TITLE** | | |  |
| Title | 1 | Identify the report as a systematic review. | 1 |
| **ABSTRACT** | | |  |
| Abstract | 2 | See the PRISMA 2020 for Abstracts checklist. | 2 |
| **INTRODUCTION** | | |  |
| Rationale | 3 | Describe the rationale for the review in the context of existing knowledge. | 3 |
| Objectives | 4 | Provide an explicit statement of the objective(s) or question(s) the review addresses. | 4 (and table 1) |
| **METHODS** | | |  |
| Eligibility criteria | 5 | Specify the inclusion and exclusion criteria for the review and how studies were grouped for the syntheses. | 4-5 |
| Information sources | 6 | Specify all databases, registers, websites, organisations, reference lists and other sources searched or consulted to identify studies. Specify the date when each source was last searched or consulted. | 5 |
| Search strategy | 7 | Present the full search strategies for all databases, registers and websites, including any filters and limits used. | Reported in protocol |
| Selection process | 8 | Specify the methods used to decide whether a study met the inclusion criteria of the review, including how many reviewers screened each record and each report retrieved, whether they worked independently, and if applicable, details of automation tools used in the process. | 5-6 |
| Data collection process | 9 | Specify the methods used to collect data from reports, including how many reviewers collected data from each report, whether they worked independently, any processes for obtaining or confirming data from study investigators, and if applicable, details of automation tools used in the process. | 6 |
| Data items | 10a | List and define all outcomes for which data were sought. Specify whether all results that were compatible with each outcome domain in each study were sought (e.g. for all measures, time points, analyses), and if not, the methods used to decide which results to collect. | 6 (and table 3) |
|  | 10b | List and define all other variables for which data were sought (e.g. participant and intervention characteristics, funding sources). Describe any assumptions made about any missing or unclear information. | 6 |
| Study risk of bias assessment | 11 | Specify the methods used to assess risk of bias in the included studies, including details of the tool(s) used, how many reviewers assessed each study and whether they worked independently, and if applicable, details of automation tools used in the process. | 6 |
| Effect measures | 12 | Specify for each outcome the effect measure(s) (e.g. risk ratio, mean difference) used in the synthesis or presentation of results. | 7 |
| Synthesis methods | 13a | Describe the processes used to decide which studies were eligible for each synthesis (e.g. tabulating the study intervention characteristics and comparing against the planned groups for each synthesis (item #5)). | Table 2 |
|  | 13b | Describe any methods required to prepare the data for presentation or synthesis, such as handling of missing summary statistics, or data conversions. | Not applicable (NA) |
|  | 13c | Describe any methods used to tabulate or visually display results of individual studies and syntheses. | 7 |
|  | 13d | Describe any methods used to synthesize results and provide a rationale for the choice(s). If meta-analysis was performed, describe the model(s), method(s) to identify the presence and extent of statistical heterogeneity, and software package(s) used. | 7 |
|  | 13e | Describe any methods used to explore possible causes of heterogeneity among study results (e.g. subgroup analysis, meta-regression). | 7 |
|  | 13f | Describe any sensitivity analyses conducted to assess robustness of the synthesized results. | NA |
| Reporting bias assessment | 14 | Describe any methods used to assess risk of bias due to missing results in a synthesis (arising from reporting biases). | NA |
| Certainty assessment | 15 | Describe any methods used to assess certainty (or confidence) in the body of evidence for an outcome. | 7 |
| **RESULTS** | | |  |
| Study selection | 16a | Describe the results of the search and selection process, from the number of records identified in the search to the number of studies included in the review, ideally using a flow diagram. | 7, Figure 1 |
|  | 16b | Cite studies that might appear to meet the inclusion criteria, but which were excluded, and explain why they were excluded. | NA |
| Study characteristics | 17 | Cite each included study and present its characteristics. | Table 5 |
| Risk of bias in studies | 18 | Present assessments of risk of bias for each included study. | Fig 3, 11 (narrative for PICO 2) |
| Results of individual studies | 19 | For all outcomes, present, for each study: (a) summary statistics for each group (where appropriate) and (b) an effect estimate and its precision (e.g. confidence/credible interval), ideally using structured tables or plots. | 8, 10, fig 3, fig4 |
| Results of syntheses | 20a | For each synthesis, briefly summarise the characteristics and risk of bias among contributing studies. | Fig 3 and table 5 |
|  | 20b | Present results of all statistical syntheses conducted. If meta-analysis was done, present for each the summary estimate and its precision (e.g. confidence/credible interval) and measures of statistical heterogeneity. If comparing groups, describe the direction of the effect. | 8, fig 3, fig 4 |
|  | 20c | Present results of all investigations of possible causes of heterogeneity among study results. | 8, 10 |
|  | 20d | Present results of all sensitivity analyses conducted to assess the robustness of the synthesized results. | NA |
| Reporting biases | 21 | Present assessments of risk of bias due to missing results (arising from reporting biases) for each synthesis assessed. | 8,11 |
| Certainty of evidence | 22 | Present assessments of certainty (or confidence) in the body of evidence for each outcome assessed. | 9, 11 (and summary of findings table) |
| **DISCUSSION** | | |  |
| Discussion | 23a | Provide a general interpretation of the results in the context of other evidence. | 18 |
|  | 23b | Discuss any limitations of the evidence included in the review. | 19, 20 |
|  | 23c | Discuss any limitations of the review processes used. | 17, |
|  | 23d | Discuss implications of the results for practice, policy, and future research. | 21 |
| **OTHER INFORMATION** | | |  |
| Registration and protocol | 24a | Provide registration information for the review, including register name and registration number, or state that the review was not registered. | 4 |
|  | 24b | Indicate where the review protocol can be accessed, or state that a protocol was not prepared. | 4 |
|  | 24c | Describe and explain any amendments to information provided at registration or in the protocol. | NA |
| Support | 25 | Describe sources of financial or non-financial support for the review, and the role of the funders or sponsors in the review. | 21 |
| Competing interests | 26 | Declare any competing interests of review authors. | 21 |
| Availability of data, code and other materials | 27 | Report which of the following are publicly available and where they can be found: template data collection forms; data extracted from included studies; data used for all analyses; analytic code; any other materials used in the review. | On request |

*From:*  Page MJ, McKenzie JE, Bossuyt PM, Boutron I, Hoffmann TC, Mulrow CD, et al. The PRISMA 2020 statement: an updated guideline for reporting systematic reviews. BMJ 2021;372:n71. doi: 10.1136/bmj.n71. This work is licensed under CC BY 4.0. To view a copy of this license, visit <https://creativecommons.org/licenses/by/4.0/>

**Deviations from the protocol**

Rationale for changes: After protocol registration, the team aligned the review with current GRADE guideline methods and the ENOVAT guideline scope. Several methodological refinements were introduced to improve decision‑relevance and transparency. Below we summarise the principal differences and their justifications.

Summary of main deviations from the registered protocol (July 2022)

1. Explicit PICO questions were developed post‑protocol

- Protocol: No explicit PICO questions were specified.
- Implemented: Ten PICOs were formulated to compare five antimicrobial classes (penicillins, tetracyclines, florfenicol, macrolides, fluoroquinolones), reflecting EMA categories and ENOVAT’s clinical decision points.
- Rationale: GRADE‑based guideline development requires tightly framed questions with pre‑specified comparators and outcomes.

1. Analytic approach: pairwise meta‑analysis instead of planned network meta‑analysis

- Protocol: Network meta‑analysis (NMA) of individual antimicrobial substances was envisaged.
- Implemented: We conducted direct, random‑effects pairwise meta‑analyses at the antimicrobial class level; no treatment ranking was produced.
- Rationale: Outcome definitions and follow‑up times varied substantially, the evidence network was sparse for several nodes. Pairwise synthesis enabled reporting absolute effects against stakeholder‑defined clinical thresholds and better supported the GRADE evidence‑to‑decision process.

1. Outcome framework refined and primary outcome operationalised

- Protocol: Outcomes were broadly listed without a GRADE‑style hierarchy.
- Implemented: We prioritised short‑term therapeutic failure; because few trials measured this directly, we used “need for re‑treatment” as a surrogate primary outcome and set a priori small/moderate/large effect thresholds using stakeholder (farmer/clinician) input. Mortality, relapse, growth, and adverse events were extracted but not pooled due to inconsistent definitions and timing.
- Rationale: To align with GRADE, we established critical vs important outcomes, incorporated patient‑important thresholds, and based imprecision on whether 95% CIs crossed these thresholds. Inconsistency was judged primarily by visual inspection rather than I2, reflecting updated GRADE guidance.

1. Search updates and date limits

- Protocol: Original search strategy as specified in the protocol.
- Implemented: We ran the protocol search on 5 July 2022 and updated it on 1 June 2025. We removed date limitations in the final update. Databases, keywords, screening processes, and inclusion/exclusion criteria were otherwise unchanged.
- Rationale: To ensure currency and completeness for the guideline package.

1. Risk‑of‑bias and certainty assessment refinements

- Protocol: General plans stated without species‑adapted tools or detailed GRADE procedures.
- Implemented: We used the modified Cochrane RoB 2.0 tool for swine RCTs (as the closest species‑relevant framework) and applied GRADE to rate certainty, explicitly downgrading for indirectness when outcomes were surrogates or poorly aligned with the decision context.
- Rationale: To employ the most appropriate available tools and make transparent, decision‑focused judgements of certainty.

Additional note Subgroup analyses by production system (feedlot vs non‑feedlot) and Mycoplasma‑positive status were added to address plausible effect modification relevant to practice; no credible effect modification was found.
